# 3D multiscale imaging of human vocal folds using synchrotron X-ray microtomography in phase retrieval mode

**DOI:** 10.1038/s41598-018-31849-w

**Published:** 2018-09-18

**Authors:** Lucie Bailly, Thibaud Cochereau, Laurent Orgéas, Nathalie Henrich Bernardoni, Sabine Rolland du Roscoat, Anne McLeer-Florin, Yohann Robert, Xavier Laval, Tanguy Laurencin, Philippe Chaffanjon, Barbara Fayard, Elodie Boller

**Affiliations:** 1 Univ. Grenoble Alpes, CNRS, Grenoble INP, 3SR, Grenoble, F-38000 France; 2 Univ. Grenoble Alpes, CNRS, Grenoble INP, GIPSA-lab, Grenoble, F-38000 France; 3 Univ. Grenoble Alpes, CHU Grenoble Alpes, CNRS, Grenoble INP, IAB, Grenoble, F-38000 France; 4 Univ. Grenoble Alpes, CHU Grenoble Alpes, LADAF, Grenoble, F-38000 France; 5 Novitom, Grenoble, F-38000 France; 6 ID19 beamline, ESRF - European Synchrotron Radiation Facility, CS40220, Grenoble, 38043 France

## Abstract

Human vocal folds possess outstanding abilities to endure large, reversible deformations and to vibrate up to more than thousand cycles per second. This unique performance mainly results from their complex specific 3D and multiscale structure, which is very difficult to investigate experimentally and still presents challenges using either confocal microscopy, MRI or X-ray microtomography in absorption mode. To circumvent these difficulties, we used high-resolution synchrotron X-ray microtomography with phase retrieval and report the first *ex vivo* 3D images of human vocal-fold tissues at multiple scales. Various relevant descriptors of structure were extracted from the images: geometry of vocal folds at rest or in a stretched phonatory-like position, shape and size of their layered fibrous architectures, orientation, shape and size of the muscle fibres as well as the set of collagen and elastin fibre bundles constituting these layers. The developed methodology opens a promising insight into voice biomechanics, which will allow further assessment of the micromechanics of the vocal folds and their vibratory properties. This will then provide valuable guidelines for the design of new mimetic biomaterials for the next generation of artificial larynges.

## Introduction

**Figure 1:**
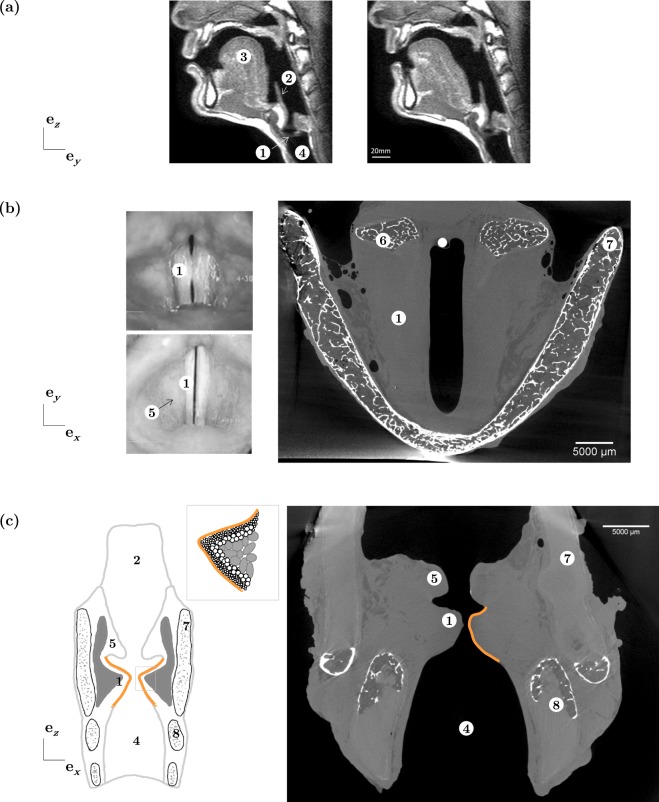
Human phonatory system. (**a**) Mid-sagittal view of the upper airways: typical IRM images obtained during production of sounds [*u*] and [*e*] respectively (male subject, source: GIPSA-lab). (**b**) Transverse view of the vocal folds: *(left) in vivo* videolaryngoscopic images obtained during a sound-pitch variation (male subject); *(right) ex vivo* X-ray microtomographic image (L_2_, *V*_*vox*_ = 25^3^ μm^3^). (**c**) Mid-coronal view of the larynx: *(left)* idealised scheme and zoom on the vocal-fold fibrous microstructure; *(right) ex vivo* X-ray tomographic image (L_3_, *V*_*vox*_ = 25^3^ μm^3^). ① Vocal fold, ② Epiglottis, ③ Tongue, ④ Trachea, ⑤ Ventricular fold, ⑥ Arytenoid cartilage, ⑦ Thyroid cartilage, ⑧ Cricoid cartilage.

Human vocal folds exhibit remarkable vibro-mechanical properties, allowing them to generate an outstanding range of sounds. These laryngeal soft tissues are in the order of 1 cm long, anteriorly and posteriorly connected to the thyroid cartilage and the two arytenoid cartilages, respectively (Fig. 1). Their self-sustained vibration is induced by pulmonary airflow and leads to phonation, *i.e*., the production of audible air pulse trains. This acoustic wave is filtered by the resonances of the vocal tract, the geometry of which is shaped by speech articulators (*e.g*., jaw, tongue, lips) to generate distinguishable voiced sounds, such as vowels and sonorous consonants. Furthermore, during this process, laryngeal intrinsic muscles drive the vocal-fold adduction and abduction, stretching, and bulging. Vocal folds behave as non-linear and coupled oscillators, able to bifurcate towards complex regimes of vibration, responding to gradual variation of control parameters^1–3^: geometrical ones (*e.g*., their distance or mean glottal width), mechanical ones (*e.g*., their stiffness) and aerodynamical ones (*e.g*., air pressure), to name a few. Their outstanding vibratory abilities are due to two major properties of the vocal-fold tissue^2,4^: (i) the ability to endure large reversible 3D deformations during phonation in the presence of numerous collisions and mechanical stresses^5^ (typically between 10–50 % stretching during a glide or intonational variations, Fig. 1(b)); (ii) the ability to vibrate with a fundamental frequency ranging from less than 50 Hz to more than 1500 Hz, which is much higher than other biological oscillators such as the heart. These properties are mainly inherited from the complex and hierarchical structure of the vocal folds and of surrounding laryngeal muscles. Adult human vocal folds are known to possess a specific lamellar structure made of several layers^6–10^, from superficial to deep:

- A stratified squamous non-keratinised *epithelium* (EP, thickness ≈50–100 μm), covered by mucus and involved in the underlying tissue protection and renewal.
- A loose connective tissue called *lamina propria* (LP, thickness ≈1–2.5 mm), made of cells and extracellular matrix (ECM) with amorphous ground substances (*e.g*., hyaluronic acid) and fibrous networks (*e.g*., collagen Type I-III and elastin, both arranged in fibre bundles of diameter ranging from 0.1 to 20 μm). The LP layer is further divided into three sublayers with distinct fibre types, densities and arrangements: the superficial one (SL), also called Reinke’s space, composed of loose fibrous components comparable to soft gelatin, the intermediate one (IL) primarily composed of elastin fibres, and the deep layer (DL) primarily composed of collagen fibres. As a whole, the LP contributes to the tissue’s “passive” biomechanical properties and to the regulation of its water content.
- The inferior thyroarytenoid muscle (M) or *vocalis* (thickness ≈7–8 mm), innervated by the inferior laryngeal nerve and responsible for the tissue’s “active” contractile properties. The inertia and stiffness of the *vocalis* vary according to the degree of contraction, to reach a given phonatory position.

Current understanding of the multiscale histological features of the vocal folds is still insufficient to make the link to their vibromechanical performance. This motivates the use of full-field 3D imaging to capture these features in human vocal folds at rest or under mechanical load:

- By contrast with other soft tissues (arteries, skin), vocal folds are not easily reachable *in vivo* by any medical 3D imaging technique (including ultrasound imaging), due to the surrounding laryngeal cartilages (Fig. 1(b,c)). Gold standard techniques used in clinics for the direct visualisation of their vibrations (high-speed cinematography, videostroboscopy)^11^ only provide partial 2D views of the vocal fold’s superior plane (Fig. 1(b)).
  Recent imaging developments of major interest include optical coherence tomography which probes a tissue sample with infrared light and uses interferometric methods to detect light reflected from up to 3 mm within the tissue^10,12,13^. Although very promising to capture the lamellar structure of the vocal folds during *in vivo* vibration, this technique is severely limited for the characterisation of the several fibrous networks in the *lamina propria* layers and the *vocalis*, due to the limited spatial resolution of about 10 μm and to the strong attenuation of light beneath the *lamina propria* layer^10,14^.
- The 3D *ex vivo* observation of excised vocal folds at the (sub-)micron scale remains a challenge. Various imaging techniques have been tested, such as micro Magnetic Resonance Imaging^15–17^, multiphoton nonlinear laser scanning microscopy (NLSM)^18–21^ and X-ray microtomography in standard absorption mode (see pretests in Fig. 1).
  Micro Magnetic Resonance Imaging is limited by its spatial resolution (even with an ultra-high magnetic field above 7T), limiting useful voxel sizes to 40^3^ μm^3^ implying that the various sublayers of the *lamina propria* cannot be observed^15^.
  NLSM offers a smaller voxel size of ≈1^3^–20^3^ μm^3^, allowing observations of the *lamina propria* sublayers. In particular, NLSM allows the fibrous networks of elastin and collagen fibres to be distinguished without exogenous staining, by the combination of two-photon autofluorescence (TPAF) and second-harmonic generation (SHG) microscopies, respectively. However, NSLM techniques exhibit three major drawbacks: (i) The TPAF and SHG signals arising from other inherently fluorescent molecules or highly ordered structures present in the vocal folds (*e.g*., SHG sources in myosin filaments, TPAF sources in epithelial cells, fibroblasts, muscle cells...)^21,22^; (ii) The long scanning time preventing 3D *in situ* observation during the deformation of samples; (iii) The depth of field which is typically limited to under 150 μm with sub-cellular resolution in such highly scattering tissues. Recently, using a longer excitation wavelength which reduces scattering probability, third-harmonic generation microscopy of porcine vocal folds has allowed images up to 420 μm deep in the superficial *lamina propria* to be acquired, albeit with a limited contrast to distinguish collagen and elastin fibres^20^. Optical clearing of vocal-fold tissue using a glycerol-based reagent is also a promising approach to reduce light scattering and thus to increase imaging depth^23,24^.
  X-ray microtomography with absorption mode and conical laboratory X-ray sources can reach sub-micron spatial resolutions (voxel size ≈300^3^ nm^3^), but suffers from two main problems: long scanning times (typically above 1 h) implying high radiation doses in the sample, and low contrast between soft biological materials (*e.g*., muscles, ECM fibres, epithelial cells which have similar X-ray attenuation coefficients) – see Fig. 1(c). These two problems can be overcome using synchrotron X-ray sources and facilities. Indeed, short scanning times can be reached^25^ (≈0.5 s per scan) with a sub-micron spatial resolution, enabling 3D *in situ* observations of samples during deformation. In addition, using phase retrieval imaging (PRI) modes, *e.g*., with the Paganin method^26^, highly contrasted 3D images of soft biological tissues can be acquired^27–29^.

Thus, following the recent studies of Vågberg *et al*.^27^ and Dudak *et al*.^28^, this work first addresses a purely technical question: (A) Does synchrotron X-ray microtomography with single phase retrieval yield sufficient contrast and spatial resolution to characterise the hierarchical structure of human vocal folds? To answer this question, 10 human larynges were scanned at various voxel sizes (≈13^3^ down to 0.65^3^ μm^3^) enabling the first *ex vivo* 3D images of human vocal folds to be obtained with this technique. The relevance and the limitations of this imaging technique (particularly at the smallest scales) are discussed by comparing the obtained 3D images with 2D optical micrographs of vocal folds prepared with standard histological stainings.

Despite the above limitations, the overall success in answering (A) allows quantitative 3D analysis of the layered structure of vocal folds, together with the geometries, orientation and the arrangement of muscular and ECM fibres inside the *vocalis* and the *lamina propria*. This, in turn, immediately poses a number of fundamental measurement questions in voice biomechanics:

(B) What are the size and spatial variation of the aforementioned layers?
(C) What is the 3D structural anisotropy of the various fibrous networks the layers are made of, as well as its spatial variation?
(D) What is the morphology of muscle and ECM fibres in their network?
(E) How do these 3D and multiscale descriptors evolve during a mechanical loading?

## Optimisation of X-ray Imaging Conditions

### Standard absorption imaging mode

A first series of scans was performed on 3 larynges (L_1_ to L_3_) using a laboratory X-ray microtomograph in standard absorption imaging mode. The spatial resolution was first set to a voxel size of 45^3^ and 25^3^ μm^3^, which is at least twice to ten times larger than the one used in standard hospital CT-scans. *A priori*, this resolution should allow the identification of the various layers of the vocal folds as well as a fibrous texture corresponding to muscle fibres or collagen/elastin fibre bundles. The other X-ray scanning parameters are listed in Table 1, but are similar to those already used on medical scanners for the radiology of *in vivo* and *ex vivo* larynges^30,31^. Typical orthogonal slices obtained with these imaging conditions are reported in Fig. 1(b,c). The external surface of the larynges is clearly identified. In addition, calcified portions of the thyroid, cricoid and arytenoid cartilages, coming from a partial ossification that starts typically at the age of 20^32,33^, are visible as lighter zones corresponding to higher attenuation – calcium exhibiting a much higher X-ray absorption coefficient than the other soft tissues of the larynges. Unfortunately, given that the chemical components of soft tissues exhibit similar absorption coefficients, within the vocal fold, there is insufficient contrast to separate fat tissues, non-ossified cartilages, muscles, *epithelium* and the inner structure of the *lamina propria*. This was not significantly improved while imaging the larynges with a synchrotron tomograph in absorption mode (Supplementary Fig. S1(a)), despite the use of a monochromatic beam that should improve contrast between chemically close materials. The 3D hierarchical vocal-fold structure could not be detected even with a voxel size of 13^3^ μm^3^, nor even when imaging cryo-preserved samples or excised vocal folds without any surrounding cartilage (Supplementary Fig. S2(a)).

**Table 1:** Scanned larynges.

Larynx name	Donated body		Alcohol immersion	Microtomographic imaging parameters				
	Gender	Age [y]	[C_2_H_6_0] [%]	*V*_*vox*_ [*μ*m^3^]	*x*_*c*_ [mm]	*U* [kV]	*I* [*μ*A]	*n*_*p*_
*L*_*1*_	*F*	*86*	*0*	*45* ^*3*^	*—*	*101*	*297*	*1 440*
*L*_*2*_	*M*	*94*	*0*	*25* ^*3*^	*—*	*100*	*300*	*3 000*
*L*_*3*_	*F*	*82*	*0*	*25* ^*3*^	*—*	*100*	*300*	*3 000*
**Larynx name**	**Gender**	**Age [y]**	**[C**_**2**_ **H**_**6**_ **0] [%]**	***V***_***vox***_ **[** ***μ*** **m** ^**3**^ **]**	***x***_***c***_ **[mm]**	***δ*** **:** ***β***	***E*** **[keV]**	***n***_***p***_
L_4_	F	90	0	13^3^	1 200	1 800	65	4 902
L_5_	F	85	0	13^3^	1 200	1 800	65	4 902
L_6_	F	89	0	13^3^	1 200	1 800	65	4 902
L_7_	M	81	0	13^3^	1 200	1 100	65	4 902
L_8_	M	89	0	13^3^	1 200	1 100	65	4 902
L_9_	F	75	[0; 30; 100]	13^3^	11 000	1 200	60	4 900
${{\rm{L}}}_{10}^{\dagger }$	F	92	direct vocal-fold dissection - see Table 2					

Italic lines refer to samples imaged with a laboratory conical X-ray source with absorption imaging mode, the others being imaged with a synchrotron source. F: female, M: male, *V*_*vox*_: voxel size, *x*_*c*_: distance between sample and camera, *U*: generator voltage, *I*: scanning current intensity, *δ*:*β*: ratio of the dispersive and absorptive aspects of the wave-matter interaction, *E*: beam energy, *n*_*p*_: number of X-ray 2D projections. ^†^Sample only dedicated to the “high” resolution imaging of the vocal-fold structure *i.e*., at a voxel size of 0.65^3^ μm^3^.

### Phase retrieval imaging mode without contrast agent

To increase the contrast between the different constituents of the vocal folds, the samples were imaged on a synchrotron X-ray source with the objective of using phase retrieval (Paganin method), at “medium” (larynx samples) or “high” (excised vocal folds) spatial resolutions. Corresponding scanning and reconstruction parameters are reported in Tables 1 and 2. The effect of the sample-to-detector propagation distance *x*_*c*_ on the phase contrast enhancement, with mid-field and far-field optical configurations was investigated. Some representative 3D images of the larynx and excised vocal folds obtained with this technique are shown in Supplementary Fig. S1(b–d).

**Table 2:** Scanned vocal-fold samples S_*i*_ extracted from larynx L_*j*_.

Vocal-fold sample					Microtomographic imaging parameters					
Name	Type	Conservation			*V*_*vox*_ [*μ*m^3^]	*x*_*c*_ [mm]	*U* [kV]	*I* [*μ*A]	*n*_*p*_	*N*
*L*_*2*_ *-S*_*1*_	*M* + *LP* + *EP*	*cryopreserved at −80 °C*			*15* ^*3*^	*—*	*100*	*100*	*1 440*	*2*
*L*_*3*_ *-S*_*1*_	*M* + *LP* + *EP*	*defrosted from −20 °C*			*12* ^*3*^	*—*	*100*	*100*	*2 496*	*2*
*L*_*3*_ *-S*_*2*_	*M* + *LP* + *EP*	*cryopreserved at −80 °C*			*12* ^*3*^	*—*	*100*	*100*	*2 496*	*1*
**Name**	**Type**	**[C**_**2**_ **H**_**6**_ **0] [%]**	**[CH**_**2**_ **0] [%]**	***δt*** **[min]**	***V***_***vox***_ **[** ***μ*** **m** ^**3**^ **]**	***x***_***c***_ **[mm]**	***δ*** **:** ***β***	***E*** **[keV]**	***n***_***p***_	***N***
L_4_-S_1_	M	30^†^	0	5	0.65^3^	40	500	19	1 995	1
L_4_-S_2_	M	30	0	5	0.65^3^	40	500	19	1 995	5
L_4_-S_3_	LP + EP	30	0	5	0.65^3^	40	500	19	1 995	4
L_6_-S_1_	M + LP + EP	30	0	4 140	0.65^3^	40	300	19	1 499	16
L_7_-S_1_	M	0	10^†^	650	0.65^3^	40	500	19	1 995	1
L_7_-S_2_	LP + EP	0	10^‡^	600	0.65^3^	22	500	19	1 995	2
**L**_**10**_ **-S**_**1**_	**M + LP + EP**	**0**	**0**	**—**	**0.65** ^**3**^	**40–400**	**300**	**19**	**1 499**	**7**
**L**_**10**_ **-S**_**2**_	**M + LP + EP**	**50**	**0**	**7 620**	**0.65** ^**3**^	**40**	**300**	**19**	**1 499**	**37**
**L**_**10**_ **-S**_**3**_	**M + LP + EP**	**70**	**0**	**5 940**	**0.65** ^**3**^	**40**	**300**	**19**	**1 499**	**34**
**L**_**10**_ **-S**_**4**_	**M + LP + EP**	**100**	**0**	**[1; 7; 13]**	**0.65** ^**3**^	**40**	**300**	**19**	**1 499**	**3**

Italic lines refer to samples imaged with a laboratory conical X-ray source. Bold lines refer to the imaged samples used for comparison with histological measurements. *V*_*vox*_: voxel size, *x*_*c*_: distance between sample and camera, *δ*:*β*: ratio of the dispersive and absorptive aspects of the wave-matter interaction, *E*: beam energy, *n*_*p*_: number of X-ray 2D projections, *N*: number of scans realised on the sample. ^†/‡^Sample kept immersed in alcohol/glue during the scan.

On the larynx at “medium” spatial resolution (hereforth defined at voxel size of 13^3^ μm^3^), phase retrieval allows adipose elements to be distinguished from other soft tissues (Supplementary Fig. S1(b,c)), which is in line with results obtained with phase retrieval imaging of human breasts^34^. However, neither the mid-field (*x*_*c*_ = 1.16 m) nor the far-field (*x*_*c*_ = 11 m) configurations were sufficient to allow clear visualisation of any multi-layered arrangement or fibrous network within the vocal folds. In the mid-field configuration, some hints of texture are discernible within the vocal tissue (see arrows in zoomed view). The far-field configuration further improves contrast (revealing several vocal-fold structural features), at the cost of detrimental reconstruction artefacts at the air-tissue interface, that spread into the sample and severely alter the image (Supplementary Fig. S1(c)).

On the excised vocal fold at “high” spatial resolution (hereforth defined at voxel size of 0.65^3^ μm^3^), a slight network-like texture is revealed within the muscular layer with the mid-field configuration (*x*_*c*_ = 40 mm), as displayed in Supplementary Fig. S1(d). However, the *lamina propria* still appears as uniform, *i.e*., without any textures related to collagen and elastin fibres. Doubling the propagation distance *x*_*c*_ to 80 mm enhances the phase contrast for the muscular layer, but also interferences at air-tissue interface. In the far-field configuration (*x*_*c*_ = 400 mm), contrast within the vocal fold is not drastically improved and details of the texture are lost due to blur.

### Phase retrieval imaging mode with contrast agents

**Figure 2:**
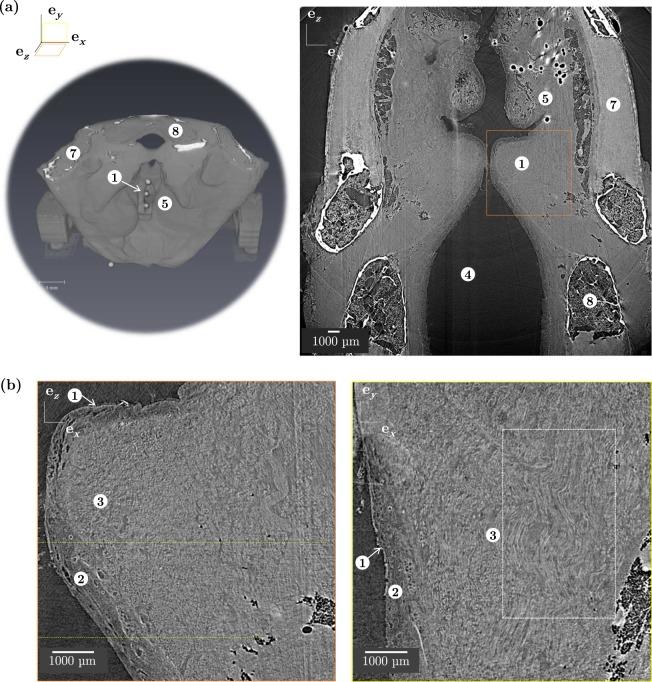
Characterisation of human laryngeal tissue’s architecture using X-ray synchrotron microtomographic PRI imaging mode and medium spatial resolution (*V*_*vox*_ = 13^3^ μm^3^). (**a**) *left:* 3D reconstruction of sample L_5_; *right:* tomographic image of a larynx vertical 2D slice, coronal plane (L_9_, [C_2_H_6_0] = 100%). ① Vocal fold, ④ Trachea, ⑤ Ventricular fold, ⑦ Thyroid cartilage, ⑧ Cricoid cartilage. (**b**) Zoom on one vocal fold and its inner fibrous architecture in coronal (orange) and perpendicular transverse (yellow) planes (L_9_, [C_2_H_6_0] = 100%). ① EP, ② LP, ③ M.

To further increase the contrast between the constituents of soft tissues, a series of excised vocal folds were first immersed into aqueous solutions of formaldehyde or ethanol and then imaged according to similar scanning parameters as above (see Table 2). As evident from the corresponding images shown in Supplementary Fig. S2, formaldehyde reduces contrast and was therefore discarded: the muscular fibrous networks of the *vocalis* now appear as a fully homogeneous material. On the contrary, the use of ethanol solutions improve contrast, in agreement with the 3D images obtained in previous studies^28,35,36^. After a 3-day immersion in an aqueous solution with 30% of ethanol (Supplementary Fig. S2(b)), the different vocal-fold sublayers can now be identified, *i.e*., the *vocalis* with well-defined cross sections of muscle fibres, the *lamina propria* with a similar but finer texture, and the *epithelium* with some saturated brightness due to sharp phase contrast at air-tissue interface. These qualitative observations are confirmed and even reinforced for an increased concentration of ethanol, as illustrated in Supplementary Fig. S2(c) in case of a 4-day immersion in an aqueous solution with 70% of ethanol. As displayed in Supplementary Fig. S2(e), immersion and scanning in pure ethanol was also attempted with the objective of measuring diffusion times. A diffusion time *δt* of 13 min was found as a minimum to identify the vocal-fold sublayers and the fibrous texture within the *lamina propria*. Given the success of pure ethanol immersion combined with phase retrieval, images of the entire larynx confined in a box filled with pure ethanol were also acquired at the “medium” spatial resolution (voxel size of 13^3^ μm^3^) and far-field configuration: Fig. 2 shows representative slices obtained in coronal and transverse planes. The wavy arrangement of muscular-fibre bundles and their orientation along the anteroposterior direction are clearly visible. In addition, although the fibrous texture within the *lamina propria* is still hardly detectable at this spatial resolution, the delineation of this layer with the *vocalis* can be well-differentiated (see Fig. 5(a)).

### Comparison with standard histological optical micrographs

**Figure 3:**
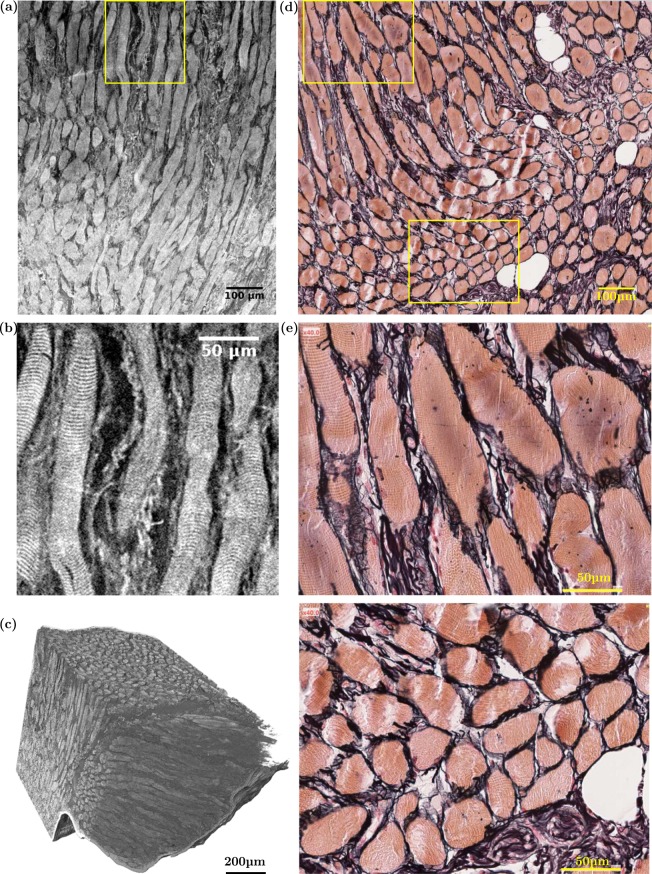
Comparison between *(left)* synchrotron high-resolution X-ray microtomographic images (PRI mode, *V*_*vox*_ = 0.65^3^ μm^3^) of the left *vocalis* fibrous network and *(right)* 2D histological photomicrographs of the right *vocalis* excised from the same larynx L_10_. (**a**) 2D coronal view of L_10_-S_3_, [C_2_H_6_0] = 70%. (**b**) Zoom on the banding patterns of the muscle fibres located within the yellow frame in (**a**). (**c**) 3D reconstruction of L_4_-S_2_, [C_2_H_6_0] = 30%. (**d**) 2D coronal view of L_10_-S_5_, prepared with Reticulin stain: Type III collagen fibres (black); muscular striated-cells or “fibres” (orange); adipocytes (white). (**e**) Zooms on the banding patterns of the muscle fibres located within the yellow frames in (**d**).

**Figure 4:**
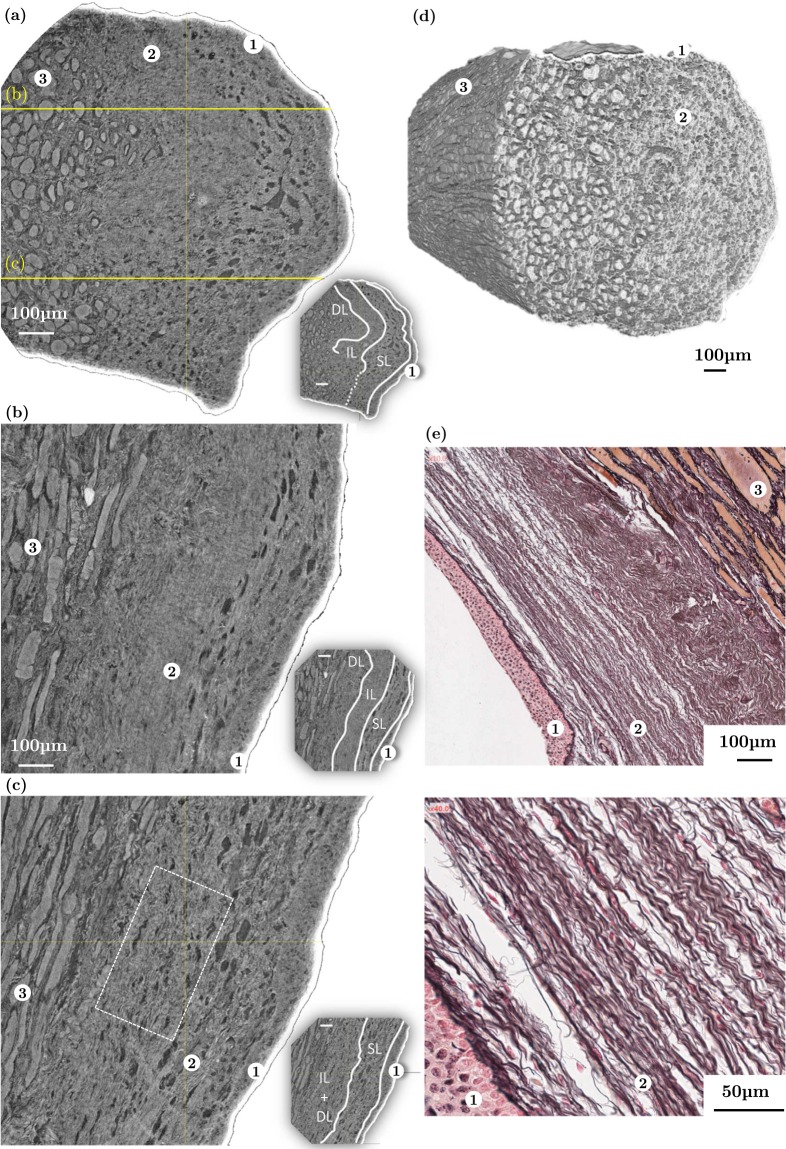
Comparison between (**a**–**d**) synchrotron high-resolution X-ray microtomographic images (PRI mode, *V*_*vox*_ = 0.65^3^ μm^3^) of the left *lamina propria* fibrous network and (**e**) 2D histological micrographs of the right *lamina propria* excised from the same larynx L_10_. (**a**) 2D coronal view of L_10_-S_3_, [C_2_H_6_0] = 70%. (**b**,**c**) Companion and orthogonal 2D transversal views. (**d**) Corresponding 3D reconstruction; (**e**) *(top)* Histological photomicrograph of L_10_-S_5_ prepared with Reticulin stain: Type III collagen fibres (black); muscle fibres (orange); stratified squamous *epithelium* (pink); *(bottom)* Zoom on the wavy arrangement of elastin and collagen bundles of fibres. ① EP, ② LP, ③ M.

Standard histological stained samples were extracted from a vocal fold. The resulting optical micrographs were compared with slices obtained on the same larynx using high resolution synchrotron X-ray tomography with phase retrieval and ethanol immersion. This comparison is shown in Figs 3 and 4 and qualitatively validates the imaging procedure. In particular, the skeletal “muscle fibres” (also called “muscle striated cells” or “rhabdomyocytes”) are usually recognised by their distinctive transverse banding patterns due to the arrangement of myofibrils. This transverse banding is detectable with both imaging techniques (Fig. 3). As shown on both images, the muscle fibres are grouped together into bundles (or *fasciculi*) and surrounded by loose connective tissue. Furthermore, multiple epithelial cell layers, closely packed, stratified, and nucleated^2,37^, are also apparent on both X-ray images and histological micrographs, thereby allowing the *epithelium* delineation (Fig. 4(a–c,e)). Moreover, the tortuous arrangement of smaller fibres which are noticeable in tomographic images between the muscle fibres of the *vocalis* and the *epithelium*, is similar to the organisation of ECM fibres in the *lamina propria* layer, as highlighted in histological views in Fig. 4 and Supplementary Fig. S3. However, such histological micrographs show that, in the investigated regions, collagen Type I, collagen Type III, and elastin fibres in the ECM are closely entangled, which makes their distinction into separate networks difficult, as is the case in nonlinear microscopy^18^. Synchrotron X-ray microtomography does not solve the issue either: we were not able to distinguish elastin fibres from collagen ones, since their X-ray absorption and phase coefficients are very close. Finally, the microstructural differences between arytenoid hyaline and elastic cartilages can also be identified with both imaging techniques (Supplementary Fig. S4). In particular, the high content of elastic fibres surrounding the chondrocytes (*nuclei*) in *lacunae* is detectable in X-ray scans of elastic cartilage.

## 3D Hierarchical Structure of the Vocal Folds

### Larynx and vocal fold geometries

Using large voxel sizes of 45^3^ and 25^3^ μm^3^, the obtained 3D images allow the definition of the outer surface of the larynx and the vocal folds (Figs 1 and 2). Using phase retrieval imaging combined with ethanol immersion, important measurements of the inner and layered structure of vocal folds can be made (see next subsections). In addition, the database of samples scanned in this work comprises four laryngeal samples (L_5_ to L_8_) scanned at rest and while being subjected to a 3D deformation resulting from cricothyroid tilt (Supplementary Figs S7 and S8). Thanks to the glass beads stuck inside the samples onto the vocal folds (see Fig. 2(a)), the longitudinal elongation *λ* of vocal folds after the larynx deformations can be estimated: depending on the tested samples, *λ* ranges from 1.05 to 1.27 with a mean value 1.13.

### Structure of the *vocalis*

**Figure 5:**
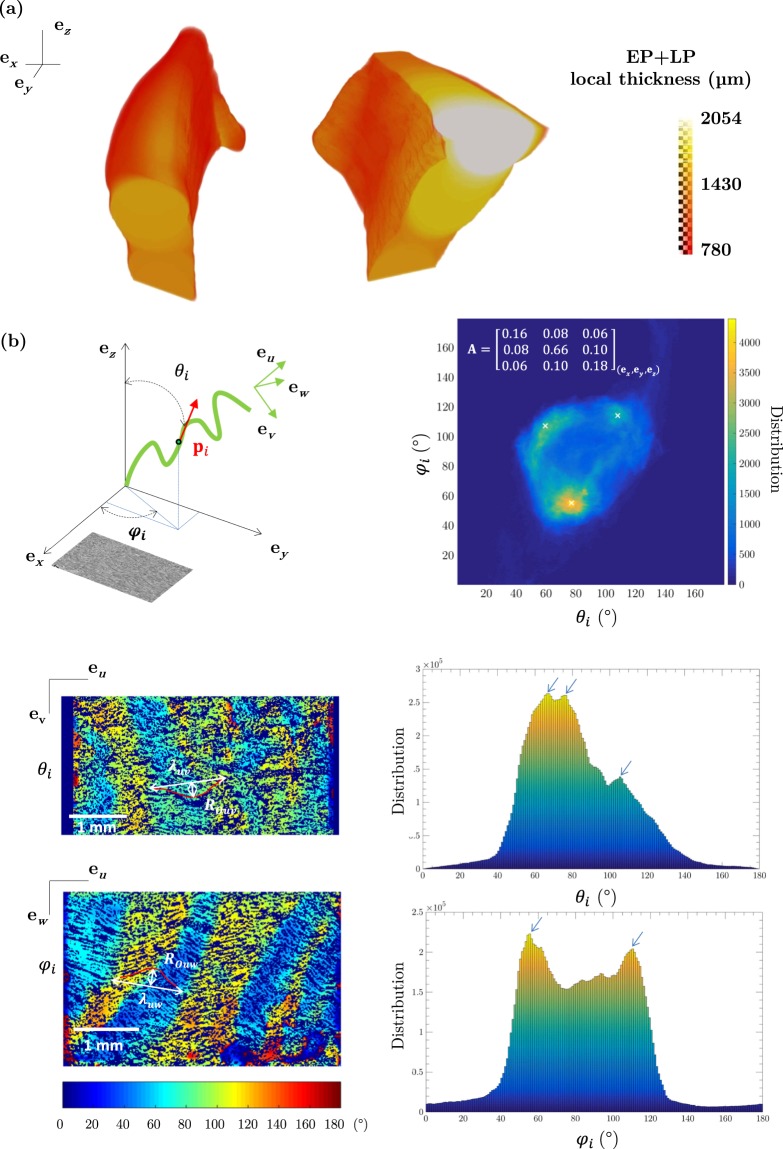
Quantification of several structural descriptors of the vocal-fold EP-, LP- and M-sublayers derived from images acquired at medium spatial resolution (voxel size of 13^3^ μm^3^) (L_9_). (**a**) 3D local thickness map of a EP+LP-layer subvolume. (**b**) 3D orientation map of the muscle fibres network in a M-layer 41.52 mm^3^ subvolume; corresponding 2D distribution of *θ*_*i*_ and *φ*_*i*_ values; illustration of their distribution on two orthogonal 2D slices of the subvolume.

**Figure 6:**
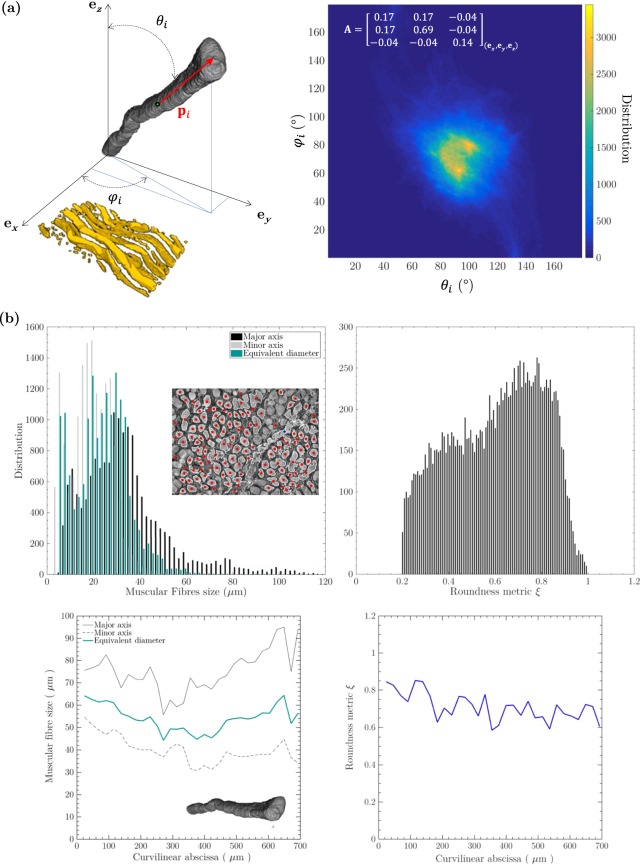
Quantification of several structural descriptors of the vocal-fold muscular layer (L_10_-S_3_) derived from images acquired at high spatial resolution (voxel size of 0.65^3^ μm^3^). (**a**) 3D orientation map of the muscle fibres network in a M-layer 0.007 mm^3^ subvolume. (**b**) Statistical dimensions and shapes of the muscular fibrous network *(top panel)* and that of an individual muscle fibre extracted from the 3D image *(middle panel)*.

In the vicinity of the *lamina propria*, the *vocalis* is constituted of well-delimited fibres, mainly oriented along the vocal-fold longitudinal direction (Figs 2 and 3). Further from the *lamina propria* (typically beyond 1 mm), other fibrous networks with perpendicular orientation are also highlighted in several muscle regions (see Fig. 3(c)). They appear at the transition between the *vocalis* and the external part of the thyroarytenoid muscle, or between the *vocalis* and the lateral cricoarytenoid muscle. In addition, some interesting results were obtained on the ethanol-preserved samples at both “medium” (voxel size of 13^3^ μm^3^, Fig. 5) and “high” (voxel size of 0.65^3^ μm^3^, Fig. 6) spatial resolutions:

#### Medium spatial resolution

Figure 5(b) shows a 3D fibre orientation map of the muscular fibrous network in the reference frame (**e**_*x*_, **e**_*y*_, **e**_*z*_), where **e**_*x*_ coincides with the mediolateral direction, **e**_*y*_, with the anteroposterior direction and **e**_*z*_, with the inferosuperior direction. The map was obtained from a subvolume of about 3.25 × 5.01 × 2.55 mm^3^ that was extracted near the *lamina propria* of larynx L_9_. The selected 3D region was characterised by a single network of muscle fibres mainly oriented along the longitudinal direction **e**_*y*_ of the fold – Fig. 2(b) shows a representative slice plotted in white within the cropped subvolume. Indeed, the mean values of the angles *θ*_*i*_ and *φ*_*i*_ are both close to 82°, the non-diagonal components of the fibre orientation tensor **A** are very small, and its component *a*_*yy*_ is much larger than *a*_*xx*_ and *a*_*zz*_. However, muscle fibres are not perfectly aligned: the standard deviations for *θ*_*i*_ and *φ*_*i*_ reach non zero values, of the order of 25° and 28°, and so the components *a*_*xx*_ and *a*_*zz*_. It is worth noting that these two components are practically equal, which means that the network of muscle fibres exhibits transverse isotropy at first order. In other words, the muscle fibres can be seen as a network with rotational symmetry with respect to the anteroposterior direction, **e**_*y*_. More precisely, three peaks emerge from the 3D orientation map (noted with white crosses on Fig. 5(b)): (*θ*_*i*_, *φ*_*i*_) = (77°, 55°); (59°, 107°); (108°, 114°). Such peaks are also highlighted once reported in cumulative histograms of *θ*_*i*_ and *φ*_*i*_ angles (noted with blue arrows). They are probably related to the 3D wavy arrangement of muscle fibres, as illustrated on representative orthogonal slices in Fig. 5(b). Please note that the variations of local *φ*_*i*_-orientations in the (**e**_*u*_, **e**_*w*_) plane as defined in Fig. 5(b) are delimited by peaks and valleys of a quasi-sinusoidal waveform, which is a characteristic of the fibrous network waviness: the corresponding spatial period and magnitude were estimated to be around *λ*_*uw*_ = 1.50 mm ± 0.15 mm and *R*_0*uw*_ = 220 μm ± 60 μm, respectively. In the orthogonal plane (**e**_*u*_, **e**_*v*_), a quasi-sinusoidal waveform is also observed, with a spatial period *λ*_*uv*_ = 1.51 mm ± 0.2 mm and an amplitude *R*_0*uv*_ = 198 μm ± 60 μm.

#### High spatial resolution

The same (but local) analysis was carried out with a subvolume of 209 × 403 × 81 μm^3^ (Fig. 6(a)) extracted from sample L_10_-S_3_ at 1.3 mm from the *lamina propria*. The corresponding fibre orientation tensor is practically identical to that found at “medium” spatial resolution (note that the *a*_*xy*_ component reaches a non negligible value due to a slight misalignment of the microstructure with respect to the reference frame). The local fibre orientation distribution is also dispersed (see the 3D orientation map in Fig. 6(a)), with standard deviations for *θ*_*i*_ and *φ*_*i*_ equal to 24° and 28°, respectively. The whole angular distribution looks rather homogeneous and centred around a single point (*θ*_*i*_, *φ*_*i*_) = (93°, 76°). Yet, two peaks emerge in the 3D orientation distribution, for (*θ*_*i*_, *φ*_*i*_) = (93°, 61°); (100°, 80°). At this spatial resolution, the analysis is sensitive to the variation of local orientation along fibres, so that both peaks can be related to their waviness, as illustrated by the segmented fibres shown in Fig. 6(a). In addition, other descriptors of muscle fibres were extracted within a subvolume of size 615 × 450 × 98 μm^3^, close to the previous one. For instance, the average surface number of muscle fibres was found to be around 380 mm^−2^, by analysing slices perpendicular to the main fibre orientation. The equivalent diameter of muscle fibres *d*_*e*_, the size of their minor and major inertia axes were computed: the corresponding distributions are plotted in Fig. 6(b). The peak value of *d*_*e*_ is 29.3 μm (median value 23.8 μm). The shape of fibres was quantified by a peak roundness metric *ξ* of 0.70 (median value 0.63), which shows that muscle fibres exhibit quasi-circular cross-sections. These results are in agreement with those obtained from a single muscle fibre of length 700 μm (Fig. 6(b)), which was isolated from the fibrous network. The mean diameter *d*_*e*_ of this fibre is 54 μm (values ranging from 44.3 to 64.3 μm) and its mean roundness metric is close to 0.71 (values ranging from 0.58 to 0.85).

### Structure of the *lamina propria* and the *epithelium*

For the *lamina propria* and the *epithelium*, the following results were obtained on ethanol-preserved samples:

#### Medium spatial resolution

The thickness of the *lamina propria* together with the *epithelium* was measured with both 2D and 3D views. In Fig. 4, regions of tissues characterised by X-ray microtomography and histological staining were positioned in the vocal-fold membranous part, at about 2–3 mm from the vocal process (arytenoid’s anterior end). Within this vocal fold (female, 92 years), the thickness in the coronal plane was measured between 530 and 930 μm from the 3D images (mean value 750 μm ± 95 μm over 50 measurements in a 3D neighboured region), and between 430 and 650 μm from the 2D histological micrographs. The 3D local thickness maps of both vocal folds were also derived on sample L_9_ (Fig. 5(a)), revealing an asymmetry between each fold, where thickness values globally varied from 780 to 2054 μm depending on the anatomical location.

#### High spatial resolution

The set of 2D and 3D micrographs related to sample L_10_-S_3_ in the mid-membranous region^38^ and displayed in Fig. 4 allows an interesting inspection of the inner and multilayered structure of vocal folds in the strongest collision zone. The slice (a), which is perpendicular to the anteroposterior direction, and its two companion and orthogonal slices (b) and (c) emphasize the *epithelium* ①, the *lamina propria* ② and the *vocalis* ③. From them, the spatial variation of the *epithelium* thickness along the vocal-fold outer surface (free edge) was estimated. For this sample, the 3D measurements yielded a thickness ranging from 30 to 80 μm, which is in line with standard 2D histological assessments (see Fig. 4(e) and Supplementary Fig. S3), and previous values reported in the literature^2,39,40^. More interestingly, X-ray microtomographic slices (a-c) also reveal sublayers of complex shapes and characteristic textures within the *lamina propria*. When these sublayers can be distinctly separated, they are delineated in Fig. 4(a–c), and interpreted according to those detectable on the 2D photomicrograph (e):

- Underneath the *epithelium*, a first sublayer is recognised (a-c, e), where the ECM fibre content is lower than in the rest of the *lamina propria*: it is characterised by large darker zones of ≈20 μm in size, aligned with the fibrous networks. This region corresponds to the superficial layer of the *lamina propria* (SL), or Reinke’s space. In the observed volumes, large variations of the SL thickness were observed, ranging between 100 and 200 μm.
- Underlying the superficial sublayer, the intermediate sublayer (IL) is also detected (a-b, e), where the ECM fibre content is higher, and the fibre waviness is far less pronounced than in the third deep sublayer (DL), located between the IL and the *vocalis*.
- The three aforementioned sublayers are clearly visible in micrographs (b, e). However, it is worth noting that this is not the case everywhere in the volume: in slices (a) and (c) for example, both IL and DL layers can hardly be separated by the naked eye.

**Figure 7:**
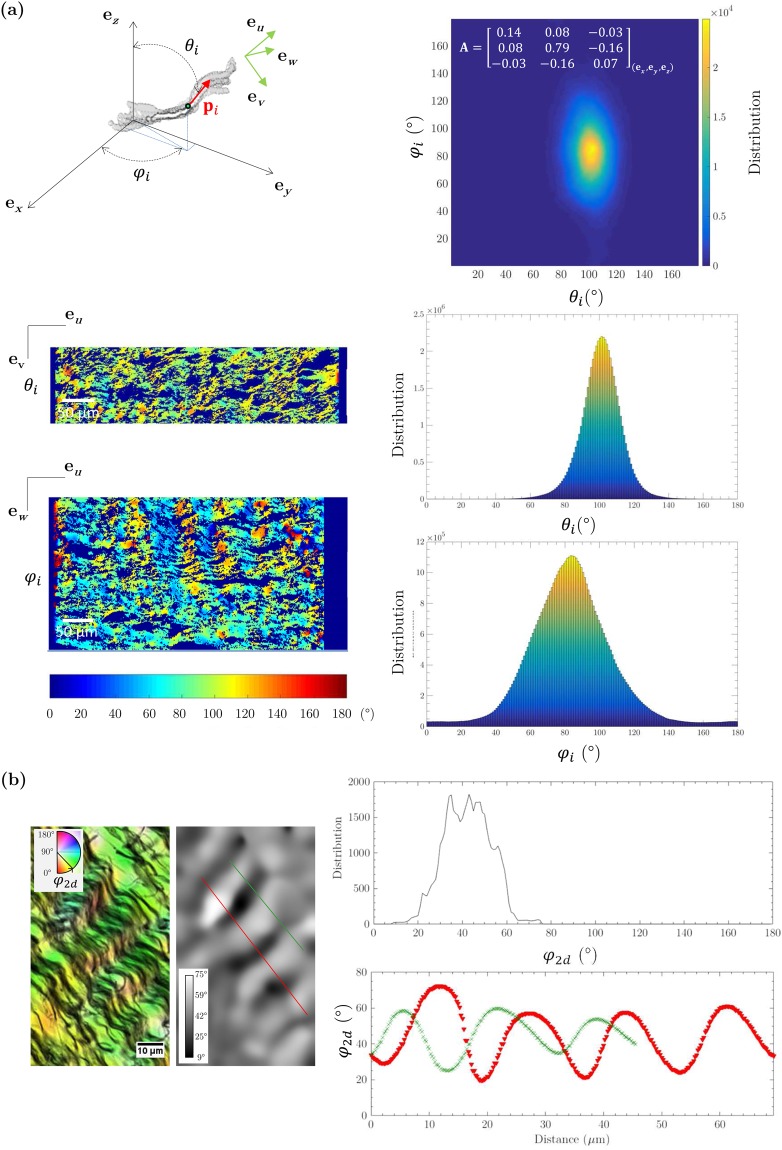
Quantification of several structural descriptors of the vocal-fold LP-layer (L_10_-S_3_). (**a**) 3D orientation map of the collagen and elastin fibrous networks in a LP-layer 0.018 mm^3^ subvolume derived from microtomographic measurements acquired at high spatial resolution (voxel size of 0.65^3^ μm^3^); corresponding 2D distribution of *θ*_*i*_ and *φ*_*i*_ values; illustration of their distribution on two orthogonal 2D slices of the subvolume. (**b**) In-plane waviness of Type III collagen fibres network, derived from histological measurements.

We further analysed the fibrous structure within the so-called “vocal ligament”, which contains both intermediate and deep sublayers of the *lamina propria*^2,41^, and is considered as the primary load-bearing portion of the vocal fold, especially at high longitudinal stretches^19^. The 3D orientation map of the network of collagen-elastin fibres together with its corresponding fibre orientation tensor **A** are given in Fig. 7(a). These data were obtained from a subvolume of 267 × 515 × 131 μm^3^ extracted from sample L_10_-S_3_ – Fig. 4(c) shows a representative slice plotted in white within the cropped subvolume. Despite the slight misalignment of the microstructure with respect to the reference frame (**e**_*x*_, **e**_*y*_, **e**_*z*_), it is fair to conclude that fibre bundles are mainly oriented along the **e**_*y*_ direction, as for muscle fibres near the *lamina propria*: the fibre bundle orientation distribution is centred around (*θ*_*i*_, *φ*_*i*_) = (102°, 85°), so that the component *a*_*yy*_ of **A** is much higher than *a*_*xx*_ and *a*_*zz*_. It is also interesting to mention that *a*_*xx*_ is twice as large as *a*_*zz*_ (the dispersion of the angular distribution is characterised by standard deviations for *θ*_*i*_ and *φ*_*i*_ equal to 12° and 25°, respectively), proving that the fibrous network of the *lamina propria* does not exhibit transverse isotropy but orthotropy: whereas muscle fibres exhibit transverse isotropy with respect to the anteroposterior direction, the fibre alignment along this direction and inside the *lamina propria* is more pronounced through the thickness of the *lamina propria* (within the plane (**e**_*x*_, **e**_*y*_)) than perpendicularly (within the plane (**e**_*x*_, **e**_*z*_)). In addition, as for muscle fibres, a quasi-periodic variation of the local orientation of fibres along the anteroposterior axis **e**_*y*_ is clearly observed on 2D slices. The spatial periods and amplitudes of the fibre waviness were thus measured in orthogonal planes (**e**_*u*_, **e**_*w*_) and (**e**_*u*_, **e**_*v*_) leading to *λ*_*uw*_ = 56.3 μm ± 16 μm, *R*_0*uw*_ = 4.6 μm ± 1.5 μm, *λ*_*uv*_ = 44.3 μm ± 15 μm and *R*_0*uv*_ = 11.3 μm ± 3 μm. Despite being slightly higher, the 3D spatial periods are consistent with the value deduced from the 2D histological micrograph shown in Fig. 7(b), *i.e*., *λ*_2D_ = 16 μm ± 1.6 μm. Finally, the 3D contourlines of several fibrous bundles of collagen and/or elastin were also detected, as illustrated in Fig. 7(a). Such bundles have a mean diameter and length around 50 μm and 400 μm, respectively. However, due to the limited contrast obtained in this zone (see Fig. 4), no threshold-based segmentation was successfully achieved to provide a statistical quantification of their dimensions and shape.

## Discussion and Concluding Remarks

Since the reference histological findings and 3D sketches on the vocal-fold multilayered arrangement provided by Hirano *et al*. in the 1970-80s^6,41,42^, very few experimental quantitative data have been collected to characterise the complex architecture of human vocal-folds, in particular at the micrometer scale. Little information is available in the literature for the structure of *vocalis* at this scale. Regarding the multi-layered structure of the *lamina propria* and the *epithelium*, the existing information is mainly derived from standard 2D histological stainings^8,37,40,41^, micro-Magnetic Resonance Imaging techniques^17^ with a restrained spatial resolution, or, in a growing number of studies, from non-linear laser scanning microscopy where some relevant quantitative structural descriptors were assessed for the ECM fibres and their networks^18–21^. In the present work, for the first time, the 3D hierarchical architecture of human vocal folds is revealed *ex vivo* by means of fast synchrotron X-ray microtomography with phase retrieval imaging mode, together with the use of a suitable contrast agent. This 3D characterisation was performed at two spatial resolutions: (i) “medium” (voxel size of 13^3^ μm^3^), with the visualisation of the vocal fold geometries, their general placement within the larynx and the identification of their layers, *e.g., vocalis*, *lamina propria* (and its sublayers) and *epithelium*; (ii) “high” (voxel size of 0.65^3^ μm^3^), with a fine examination of muscular and ECM fibres as well as fibrous networks. Comparing results of the aforementioned studies with those obtained here, some answers to the technical and fundamental questions (A-E) listed in the Introduction can now be discussed:

### Relevance of synchrotron X-ray microtomography

To answer question (A), a methodology was elaborated to prepare vocal-fold samples with suitable conditions of tissue conservation, and to optimise the optical settings for scanning. The 3D imaging protocol was validated by comparing 3D images with standard 2D histological micrographs. A high degree of accordance was found between both techniques from a qualitative standpoint. Quantitatively, however, some differences were found, *e.g*., for the thickness of the *lamina propria* or for the waviness of its fibres. Although small, these discrepancies may be mainly ascribed to the manipulations to which the tissue is subjected during the protocol for histological staining, *e.g*., pre-deformation of samples, dehydration and possible induced shrinkages, warming, paraffin-embedding, microtomy artefacts... This point undoubtedly constitutes a strength of 3D X-ray imaging. Furthermore, compared with histological staining or other advanced 3D imaging techniques such as multiphoton microscopy^18–22,43^, the other interesting advantage of X-ray 3D imaging is the possibility (i) to get high resolution and in depth 3D images of soft tissues (ii) with very fast scanning times (here the scans were 1–2 min long). However, the technique exhibits three main limitations that should be overcome for future observations. First, the use of ethanol was necessary in order to enhance phase contrast: its impacts on the mechanical behaviour of vocal fold tissues must be assessed before performing mechanical tests with 3D *in situ* observations. Note that the relatively short duration of fixation of 13 min – the minimum for the fibrous texture of the *lamina propria* to be observed – should limit the impact on the mechanical behaviour of the tissue. Second, we experienced difficulties in discriminating collagen fibres from elastin fibres, since both their absorption and phase coefficients are very close. A first possible solution to overcome this problem consists in selectively digesting a target protein from the vocal-fold tissue, as previously done in the case of arterial walls^44^. Another solution could be to use chemical markers able to track one specific fibrous protein^29,45^. Last, we were not able to properly analyse the morphology of individual ECM fibres: this challenging task could be achieved by further increasing the spatial resolution of the images^46^.

### Size and spatial variation of the vocal fold (sub)layers

Regarding question (B), the thickness of the *lamina propria* and the *epithelium* is known to vary with gender and age^37,41,47,48^. The local 3D thickness map displayed in Fig. 5(a) demonstrates that it also varies with the anatomical location within the vocal-fold tissue, being the thickest at the midportion and becoming thinner towards the anterior and posterior ends, in agreement with previous 2D studies^41^. Besides, the qualitative division of the *lamina propria* into a superficial sublayer (SL) and a “vocal ligament” (IL + DL), as illustrated in Fig. 4(a–c), is in line with the typical distribution reported by Gray *et al*.^37^, *i.e*., with a SL-layer ranging between 25% to 35% of the initial depth of the *lamina propria*. However, even in the mid-membranous region of the vocal folds, the 3D images show that this trilamellar structure is not always observable. More precisely, we found that the intermediate sublayer (IL), which is marked by a dense network of straighter ECM fibres, may be confined to a region likely to endure the highest collision stresses during phonation^38,49^. This heterogeneous distribution in the vocal ligament is consistent with the structural measurements previously achieved by Klepacek *et al*.^17^ using micro-MRI and plastination methods. It also supports the hypothesis that regions exposed to higher stresses in the vocal tissues are characterised by a higher density of fibrous proteins produced to strengthen the ECM^37,50^.

### 3D structural anisotropy of fibrous networks in the *lamina propria* and the *vocalis*

Within the vocal ligament, Kelleher *et al*.^19^ estimated the 2D orientation distribution function of collagen fibres which was centred along the anteroposterior axis. We computed the corresponding 2D second-order fibre orientation tensor:

1 $${\bf{A}}={[\begin{array}{cc}0.15 & 0\\ 0 & 0.85\end{array}]}_{({{\bf{e}}}_{x},{{\bf{e}}}_{y})}$$

the components of which are very close to those we obtained in (**e**_*x*_, **e**_*y*_) for the 3D fibre orientation tensor reported in Fig. 7(a). Thanks to the 3D images, it is now possible to extract 3D properties of the fibre orientation in the vocal, and thereby to answer question (C): at the first order, it neither exhibits a 2D layered symmetry, nor transverse isotropy, but orthotropy with a pronounced major orientation along the anteroposterior direction, and minor and intermediate orientations along the inferosuperior and mediolateral directions, respectively. It is worth noting that this result does not fit with the 3D schematic fibrous arrangement proposed by Madruga de Melo *et al*.^8^ for the *lamina propria*: the fibrous architecture these authors suggested exhibits orthotropy without any privileged direction, while it is clearly highlighted here along the anteroposterior direction. This information is also important to build relevant micromechanical models for the *lamina propria*^19,47,50,51^, that are able to accurately reproduce the biomechanical properties of vocal tissues in multiple directions close to those encountered *in vivo*. For example, Kelleher *et al*.^19,50^ showed that both the degree and the magnitude of the vocal ligament anisotropy strongly impact the predictions of its fundamental frequency of vibration. Also, new information was obtained regarding the structural anisotropy of muscle fibres in the *vocalis*. The resulting 3D fibre orientation distributions demonstrate that muscle fibres are mainly aligned along the anteroposterior axis, with a transverse isotropy texture. Here again, this information is important to fully characterise and model the mechanics of the *vocalis* under realistic physiological loadings^52,53^. In particular, Böl *et al*.^53^ showed that such an anisotropic property implies different stress responses of the tissue under the applied compression modes, which is critical for the better understanding of vocal-fold collision.

### Morphology of ECM and muscle fibres

Regarding question (D), Miri *et al*.^18^ quantified the wavy shape of collagen fibres from three *lamina propria* (65 year-old female larynx, 55- and 68-year-old male larynges). The average period *λ*_0_ was found to be around 35 μm (values ranging from 18 to 48 μm), while the average amplitude *R*_0_ was found to be equal to 6 μm (values ranging from 2.6 to 8.4 μm). The waviness parameters we estimated from X-ray microtomographic images are consistent with the aforementioned values, although lying in the highest range. Furthermore, regarding muscle fibres in the *vocalis*, prior works in the 1980s reported a typical equivalent diameter of 32 μm for (white) fast-twitch fibres and 28 μm for (red) slow-twitch fibres, based on 2D microscopic differentiation^54^. This is in line with the equivalent circular diameter found in the present study at the scale of the 3D network. These structural data are very helpful to understand and model the mechanics of vocal folds.

### **Towards mechanical tests with 3D*in situ*observations**

At “medium” spatial resolution, a preliminary collection of 3D images was acquired for larynges subjected to anatomical placements close to those occurring during a typical phonatory cycle. This constitutes an important database which can be used either to study the larynx and vocal fold anatomy^31,55^ or to simulate the mechanics of vocal folds^56,57^. For example, the vocal fold elongation *λ* which was measured after the laryngeal tilt is consistent with the recent measurements obtained by Lagier *et al*.^55^. Using 10 excised human larynges prepared and deformed in close conditions (body ages from 71 to 92, freezing in 0.9% saline, double adduction), these authors reported *λ*-values ranging from 1.05 to 1.28 (mean value 1.12). Finally, with a scan duration of 1–2 min, it is worth mentioning that the adopted methodology is very promising to further track, at “high” spatial resolution, the deformation and the rearrangement of muscle and ECM fibres during mechanical load, thereby answering question (E). Such information is crucial to gain an in-depth understanding of the link between the micromechanics of vocal-fold tissues and their unique vibratory performances. Although left static in the present study, a part of the scanned samples were clamped inside a micropress mounted in the imaging set-up, proving the feasibility of future *in situ* tension-compression tests.

## Methods

### Samples preparation

The experiments were carried out with 10 *ex vivo* and healthy human larynges, referred to as L*i*, *i* ∈ [1, …, 10]. Samples details are given in Table 1. Anatomical pieces were excised from donated bodies within 48 h *post-mortem* at the Laboratory of Anatomy of the French Alps (LADAF - UGA, Grenoble Hospital Univ.). All but one larynx (*fresh* larynx L_10_) were preserved by freezing (−20 °C) up to X-ray scanning protocol and after. Experimental protocols were approved by the Bioethics Committee of the General Directorate for Research and Innovation (DGRI - French Research Ministry) and the LADAF. All experiments were conducted as regulated by the French ethical and safety laws in the frame of Body Donation (voluntary donation, made by the donor during his lifetime, after written and informed consent), in accordance with the ESRF Safety Office for Biology and Biochemistry and 3SR Lab policy. Samples were prepared following several protocols developed for testing and improving the microstructure exploration of the vocal-fold tissue, first with entire larynges, then on dissected vocal-fold samples:

#### Experiments on larynges

**Figure 8:**
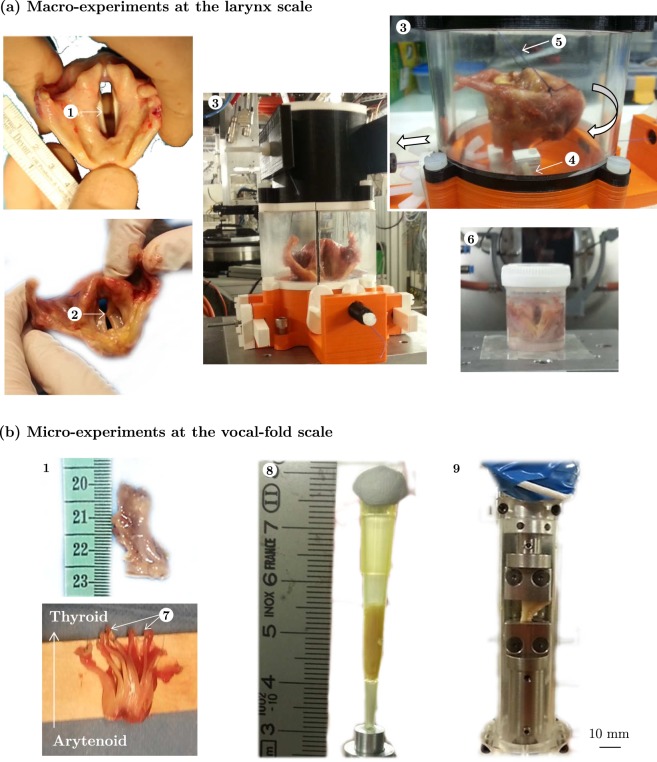
Experimental set-ups developed for X-ray microtomography characterisation of the vocal-fold tissues (**a**) within the preserved laryngeal structure and (**b**) once dissected. ① Vocal fold, ② Stitch between vocal processes, ③ Macro-mechanical set-up, ④ Wooden pike, ⑤ Wires blocking thyroid’s branches, ⑥ Set-up for sample L_9_, ⑦ Dissection procedures for samples L_*i*_-S_*j*_, ⑧ Pilot set-up, ⑨ Micro-mechanical tension device.

Larynx samples L_1_ to L_9_ were used with experimental details given in Table 1. Before any manipulation, each sample was defrosted 20 min in water (T ≈ 20 °C), then placed in a confined and fully-hydrated set-up, to reproduce realistic laryngeal phonatory placement and to control *vocalis* muscle stretching^55^ (Fig. 8a). The set-up is made of 3D-printed PLA pieces and two PMMA half-cylinders forming a sealed transparent chamber (inner diameter of 8 cm, wall thickness of 5 mm). It was designed to mimic the rocking movement of the thyroid cartilage forward and backward on the cricoid cartilage, which elongates the vocal folds *in vivo*. Mounting a larynx in it comprised several steps: (i) a stitch was made between the vocal processes of the arytenoid cartilages using a 5/0 surgical wire (Polysorb^®^), so as to adduct the arytenoid cartilages and held the vocal folds close together in a phonatory-like position^55^ (Fig. 8(a.2)); (ii) a stiffer 3/0 surgical wire was also fixed around the cricoid cartilage anterior part, passing through the thyrocricoid membrane, and linked to a manual activator of the cartilages’ rocking motion; (iii) a wooden pike was introduced trough the thyroid cartilage and fixed into the containment device to ensure a reference static position (Fig. 8(a.4)); (iv) Glass beads (1 mm in diameter) were stuck on larynx cartilages using tissue glue (PeriAcryl^®^90, Surgibond^®^) so as to add 3D positions of reference and help to identify the laryngeal structures during X-ray data collection. Such markers also allowed to measure the vocal fold elongation during the rocking movement of the larynx; (v) the larynx was then placed into the containment device, blocking the thyroid cartilage thanks to the wooden pike and to additional wires stitched through its both ended branches (Fig. 8(a.5)); (vi) the whole device was kept opened in an airtight chamber which was regulated at proper hygrometric conditions (100% RH, achieved with a humidifier Fisher & Paykel HC150). Samples L_1_ to L_8_ were subjected to steps (i-v). For sample L_9_, the larynx was directly confined in a sealed box filled with a dilute aqueous solution of ethanol (Fig. 8(a.6)) the concentration of which, [C_2_H_6_0], was set to 0, 30 and 100%.

#### Experiments on dissected vocal folds

To enable 3D imaging at micrometer-scale resolution, vocal-fold samples were dissected from six laryngeal specimens L_*i*_ (*i* = 2, 3, 4, 6, 7, 10 – see Table 2). All but one larynx (*fresh* larynx L_10_) were already exposed to X-ray radiation prior to dissection. They were cut along the anteroposterior direction with portion of thyroid and arytenoid cartilages, respectively (Fig. 8(b.1)). The dissection procedure yielded to a number *n*_*i*_ of vocal-fold samples derived from each larynx L_*i*_, labelled as L_*i*_-S_*j*_ (*j* ∈ [1, …, *n*_*i*_], Fig. 8(b.7)). As indicated in Table 2, samples were constituted either with only the muscular layer (M), or with several layers (LP+EP, M+LP+EP). It is worth noting that for the specific case of fresh larynx L_10_, sample L_10_-S_3_ was cut into the region of highest collision, *i.e*., close to the narrowest glottis^38^. Several conditions of tissue conservation were used: (i) defrosting after preservation at −20 °C; (ii) cryopreservation at −80 °C using dry ice; (iii) pre-immersion in a dilute aqueous solution of ethanol with concentrations [C_2_H_6_0] ranging from 30% to 100% and diffusion time *δt* ranging from 1 min to more than 5 days (for diffusion times larger than 24 h, the immersion was achieved in successive baths of increasing alcoholic concentrations^36^, *i.e*., 30%, 50% and 70%); (iv) pre-immersion in a 10%-formaldehyde (CH_2_0) solution. Two different containment procedures were tested. Vocal folds dissected from L_4_ and L_7_ were released from their cartilaginous ends and confined into a conic container (maximal inner diameter 5 mm and wall thickness 1 mm) sealed at its boundaries and optionally filled with glue or alcohol (Fig. 8(b.8)). Vocal folds dissected from L_6_ and L_10_ were mounted in a dedicated tension-compression micro-press^58^ (Fig. 8(b.9)), with a chamber regulated at proper hygrometric conditions (100% RH), to explore the feasibility of future *in situ* tensile tests. Typical dimensions of the scanned samples were within 10 mm × 5 mm × 5 mm (Fig. 8(b.9)).

### X-rays microtomography

#### Laboratory X-ray microtomography

Pre-tests were performed with a standard laboratory X-ray source (RX Solutions, 3SR Lab, Grenoble, France) equipped with a conical polychromatic and divergent beam (Hamamatsu L12161-07 source), allowing absorption imaging mode. Corresponding imaging parameters are reported in Tables 1 and 2 (italic lines)^30,31^. The scans were obtained with a number *n*_*p*_ of X-ray 2D radiographs onto a 1914 × 1580 pixel^2^ Varian flat panel detector, leading to a voxel size *V*_*vox*_ (varying from 12^3^ to 45^3^ μm^3^). Samples were exposed to a 360° rotation with respect to the X-ray source (step angle 360°/*n*_*p*_ varying from 0.07° to 0.25°), with an exposure time of 125 ms (respectively 400 ms) per radiograph for experiments on the whole laryngeal structures (respectively on dissected vocal tissues). To restrain the noise, an average of 6 radiographs per 2D image was used.

#### Synchrotron X-ray microtomography

Most of samples were imaged on the ID19 beamline of the European Synchrotron Radiation Facilities (ESRF, Grenoble, France), allowing advanced imaging possibilities thanks to: (i) a high photon flux in a homogeneous, parallel, monochromatic and highly coherent beam; (ii) the recording of phase images obtained by adjusting the sample-to-detector propagation distance, *x*_*c*_, using single phase retrieval imaging mode (Paganin)^26^. The interaction of X-ray with matter is generally described by the complex refractive index of the sample, n = 1 − *δ* + *iβ*, where *δ* is related to the phase-shift effects of the X-ray waves induced by the sample, and *β* determines their attenuation^59,60^. The term *β* is retrieved by recording the absorption images, *i.e*. for *x*_*c*_ = 0, while *δ* can be retrieved using a single propagation according to Paganin’s work, provided that the ratio of the dispersive and absorptive aspects of the wave-matter interaction, *δ*:*β*, is known. The chosen imaging parameters are reported in Tables 1 and 2 for experiments achieved at “medium” and “high” spatial resolutions, respectively (*i.e*., at voxel size of 13^3^ and 0.65^3^ μm^3^, respectively)^36,61–63^. Corresponding optical set-ups are detailed thereafter:

#### Experiments on larynges

For these experiments, two optical set-ups were tested:

- For laryngeal samples L_4_ to L_8_, a first series of scans was achieved using the mechanical set-up shown in Fig. 8. Two extreme phonatory positions were acquired sequentially for each sample: at rest in a first time, and at maximal macroscopic stretch of the vocal folds achieved by cricothyroid approximation in a second time. The X-ray beam was adjusted using filters (2.8 mm Al, 0.14 mm Cu), an average energy *E* of 65 keV (*I* = 200 mA) and a wiggler gap of 95 mm. The transmitted beam was converted into visible light by a LuAg scintillator (thickness 500 μm), and recorded using a CCD camera (FReLoN-2K, 2048 × 2048 pixel^2^ chip, 14-bit dynamic range, FTM mode)^64,65^. The combination of the optics, using a sample-to-detector distance *x*_*c*_ = 1.2 m in average, with the pixel size of the CCD (14^2^ μm^2^) allowed to work at “medium” spatial resolution, *i.e*. with an effective voxel size of 13^3^ μm^3^. 2D projections were collected according to the half-acquisition mode to obtain a 3D field-of-view of maximal size 3587 × 3587 × 600 voxels for each rotational acquisition. 4900 projections were acquired over the 360° rotation of samples. The beam exposure time was 100 ms per projection. Three rotational acquisitions were necessary to fully cover the whole vocal-fold tissue along its longitudinal axis (anteroposterior direction). These acquisitions were taken sequentially with an overlap of 127 slices (1.60 mm) from the angle of the thyroid cartilage. In the end, the scan duration was of 10 min and the total acquisition time about 1 h to scan both phonatory positions per larynx.
- For sample L_9_, the optical set-up was modified so as to raise the sample-to-detector distance up to *x*_*c*_ = 11 m and thereby maximise the phase-contrast effect^61,62^, by placing the sample in the ID19′s monochromator hutch (allowing a propagation distance up to 14 m). Filters were updated (2.8 mm Al, 0.7 mm Cu, 0.28 mm Au), the average energy was kept at around 60 keV, and the wiggler gap fixed at 61 mm. Placed in a single static geometry at rest, sample L_9_ was scanned in this optical “far-field” configuration yielding to a volume of 3706 × 3706 × 600 voxels (other collection parameters being as above).

#### Experiments on dissected vocal folds

For these experiments, the optical set-up was changed to work at “high” spatial resolution, *i.e*. at a voxel size of 0.65^3^ μm^3^. In addition, a GGG10 scintillator and a PCO Edge 4.2 camera (SN 62000031, 2048 × 2048 pixel^2^ chip, no binning, FFM mode) were used, allowing fast and highly contrasted imaging. The acquisition parameters were characterised by a 19 keV beam energy, an undulator gap of 21 mm, a sample-to-detector distance *x*_*c*_ = 40 mm in average, and a beam exposure time of 20 ms per radiograph. Several acquisitions were taken sequentially to cover the full height and width of the vocal-fold sample, with a field of view displaced by 1 mm-step in longitudinal and transversal directions, yielding to an overlap of 300 μm. In the end, the scan duration was within 1–2 min and the total acquisition time about 1 h 35 min per sample.

### Image processing

After reconstruction of the scans using Paganin’s method for phase retrieval coupled with filtered back-projection^26^ and removal of tomographic artefacts such as rings, the resulting 3D and greyscale images were analysed using Fiji®, Avizo® and Matlab® routines. Some of them were automatically segmented using standard smoothing and thresholding algorithms implemented in these software, to get meaningful 3D views (Figs 2(a), 3(c) and 4(d)) and to analyse the vocal-fold structure in detail (Figs 5(b), 6 and 7). In some cases, this was not possible. Thus, to pursue the quantitative analysis, manual image thresholding was carried out using Fiji® and a graphical tab (Wacom Cintiq 22HD touch). For example, the geometries of two vocal folds and the sublayers of their *lamina propria* were tracked on images acquired at “medium” spatial resolution (voxel size of 13^3^ μm^3^), as shown in Fig. 5(a). Similarly, some individual muscle fibres and ECM fibre bundles were extracted from the “high” resolution images (voxel size of 0.65^3^ μm^3^), to analyse their geometry (Figs 6(a) and 7(a)). Then, various operations were realised, to extract from these 3D images some relevant qualitative information and quantitative descriptors:

#### Vocal-fold elongation

From the segmented images of the larynges acquired before and after the rocking movement of the thyroid cartilage into the dedicated set-up (Fig. 8(a)), the local elongation of the vocal folds *λ* was measured thanks to the glass beads stuck on them. For that, beads were easily isolated from the rest of the larynx using thresholding algorithms (Fiji®), as illustrated in Supplementary Figs S7 and S8. The positions of the bead centres of mass were then detected with the 3D Particle Analyser plug-in of Fiji®. Their relative distance *l*_0_ and *l* was calculated in the non-deformed and deformed configurations respectively, to estimate the vocal-fold elongation *λ* = *l*/*l*_0_.

#### Thickness of the lamina propria

A crop of the 3D image of larynx L_9_ was achieved in order to focus on the *lamina propria* structure at the millimetre scale. After manual segmentation (see above), the spatial thickness of the extracted *lamina propria* was computed using the Local Thickness plug-in of Fiji®^66,67^.

#### Multiscale orientation of fibres

The global 3D structural anisotropy of the muscle fibres was estimated from the greyscale image of larynx L_9_ acquired at “medium” spatial resolution (voxel size of 13^3^ μm^3^), thanks to the fibrous textures detected in the *vocalis*. Notice that this operation was not possible in the *lamina propria*, where no well-defined fibrous texture was observed at this scale. Thanks to the 3D images recorded at “high” spatial resolution (voxel size of 0.65^3^ μm^3^), the local 3D structural anisotropy of muscle fibres in the *vocalis* was also estimated, as well as the global 3D structural anisotropy of ECM fibres in the *lamina propria*. Whatever the tissue and spatial resolution, the following processing route was used for each considered Region Of Interest (ROI) – see Supplementary Figs S5 and S6: First, to enhance the grey-level gradients of the fibrous phases, 3D images were subjected to a coarse thresholding process, and a series of 3D smoothing and morphological operations (median filter, hole filling, opening/closure sequences). Second, the 3D Euclidean distance map was calculated in the considered ROI. Therewith, a home-made Matlab® code was used (i) to compute the gradients of grey levels in the as-treated ROI with a centred finite difference scheme, and (ii) to build from these gradients 3D structure tensors^68^ for the *N* voxels *i* of the ROI. For that purpose, the lateral size of the Gaussian-shaped windows used to compute structure tensors was adapted to the size of the objects to be characterised: 21 voxels (273^3^ μm^3^) for the muscle fibres of Fig. 5(b), 17 voxels (11^3^ μm^3^) for those shown in Fig. 6(a), 21 voxels (13^3^ μm^3^) for the ECM fibres of Figs 7(a) and 7 pixels (2 μm) for those shown in Fig. 7(b). Then, the discrete 3D Orientation Distribution Function (ODF) of the minor eigen- and unit vectors **p**_*i*_ of the structure tensors was built. Such vectors, locally parallel to the fibres, can be expressed in the reference frame of the ROI (**e**_*x*_, **e**_*y*_, **e**_*z*_), as follows:

2 $${{\bf{p}}}_{i}=\,\sin \,{\theta }_{i}\,\cos \,{\phi }_{i}{{\bf{e}}}_{x}+\,\sin \,{\theta }_{i}\,\sin \,{\phi }_{i}{{\bf{e}}}_{y}+\,\cos \,{\theta }_{i}{{\bf{e}}}_{z},$$

where 0 ≤ *θ*_*i*_ ≤ 180° is the angle between **p**_*i*_ and **e**_*z*_, and where 0 ≤ *φ*_*i*_ ≤ 180° is the angle between the projection of **p**_*i*_ in the (**e**_*x*_, **e**_*y*_) plane and **e**_*x*_. The first non-zero moment of this ODF, *i.e*. the 3D second-order fibre orientation tensor **A**^69^, was derived as a compact and meaningful descriptor of the fibre orientation in the ROI:

3 $${\bf{A}}=\frac{1}{N}\sum _{i=1}^{N}{{\bf{p}}}_{i}\otimes {{\bf{p}}}_{i}\mathrm{.}$$

#### Waviness of fibres

As shown in the 3D images (Figs 3(c), 4(d) and 6(a)), muscle and collagen/elastin fibres exhibit a quasi-periodic wavy shape. Thus, in a given ROI, we scrutinised two sets of orthogonal slices comprising the main fibre direction **e**_*u*_, *i.e*. slices within the planes (**e**_*u*_, **e**_*v*_) and (**e**_*u*_, **e**_*w*_) as sketched in Fig. 5(b): **e**_*u*_ is defined as the main eigenvector associated to the major eigenvalue of the fibre orientation tensor **A**, **e**_*v*_ and **e**_*w*_ being eigenvectors related to the second and third eigenvalues respectively. Thereby, the spatial periods *λ*_*uv*_ (respectively *λ*_*uw*_) as well as the magnitudes *R*_0*uv*_ (respectively *R*_0*uw*_) of the quasi-periodic shape of fibres were estimated. This was done by manually clicking at least 50 data per descriptor.

#### Sections of muscle fibres

Two methods were used to analyse the size and shape of the sections of muscle fibres. Both of them used “high” resolution 3D images of the *vocalis* (voxel size of 0.65^3^ μm^3^). The first method consisted in analysing slices perpendicular to the mean orientation of muscle fibres. As depicted in the inset of Fig. 6(b), the edges of muscle fibres were detected using an edge detection subroutine (Matlab®). Then, the length *P* of each fibre’s edges was estimated, together with the surface *A* of the closed area they defined, the equivalent fibre diameter ${d}_{e}=\sqrt{4A/\pi }$, the roundness of fibre $\xi =4\pi A/{P}^{2}$ and the position of their centre of mass *x*_*G*_. During this process, muscle fibres that were misaligned, *i.e*., with the tangent trajectory of their centre of mass *x*_*G*_ too far from the main orientation of muscle fibres, were discarded. The same quantities were measured with the second method, which was applied along the whole cross sections perpendicular to the centreline of the muscle fibre that was manually extracted from its network (see bottom panels of Fig. 6(b)).

### Histological analyses

A standard histological campaign with staining and 2D optical microscopy was also conducted to compare and validate the 3D images obtained with high-resolution X-ray synchrotron tomography. To this end, the vocal-fold tissue of *fresh* larynx L_10_ was characterised with both imaging techniques, using two excised samples from the right and left vocal folds, respectively for tomography (L_10_-S_3_ in Table 2) and for standard 2D histology. The latter sample, noted L_10_-S_5_, was first fixed directly after body excision by two successive baths containing a standard solution of 4% neutral buffered formalin for 1 h each (no freezing phase). Water was then removed using a series of ethanol baths (progressive concentrations of 70%, 80%, 95%, 100%, 100%, 100%; bath duration 1 h) and the sample was cleared with xylene, miscible with paraffin (two baths of 1 h). The tissue was infiltrated with liquid paraffin at 50 °C (two baths of 1 h) and then kept in a cold atmosphere (4 °C). It was then cut into 3 μm sections using a microtome, floated on a warm water bath to remove wrinkles, picked up on a glass microscopic slide and then sequentially plunged into xylene, alcohol and water baths, thereby reversing the embedding process so as to get the paraffin wax out of the tissue, and allow water-soluble dyes to penetrate the sections. Finally, different stains were chosen to enhance contrast under optical microscopy (resolution 0.2 μm): Hematoxylin-Eosin-Saffron (HES), Reticulin (modified Gordon and Sweets stain, Reticulum II staining kit, Roche) and Masson Trichrome were used to reveal both Type I and Type III collagen fibres (the latter being also called “reticular” fibres), whereas an Elastic stain allowed elastin fibres to be emphasised (see Supplementary Fig. S3).

## Electronic supplementary material

Supplementary information

## Data Availability

The datasets generated and/or analysed during the current study (≈1.5 To) are available from the corresponding author on request. An open access upon the ESRF data portal is also possible, according to the ESRF data policy (https://www.esrf.eu/datapolicy)﻿.
