# Supplementary material for: 3D multiscale imaging of human vocal folds using synchrotron X-ray microtomography in phase retrieval mode
Source: Sci Rep. 2018 Sep 18;8:14003. doi: 10.1038/s41598-018-31849-w (PMC6143640; doi:10.1038/s41598-018-31849-w)
Supplement: Supplementary file 1 — Supplementary information [file 41598_2018_31849_MOESM1_ESM.pdf]

3D multiscale imaging of human vocal folds using synchrotron X-ray microtomography in phase retrieval mode

Lucie Bailly[1,*], Thibaud Cochereau[1,2], Laurent Orgéas[1], Nathalie Henrich Bernardoni[2], Sabine Rolland du Roscoat[1], Anne McLeer-Florin[3], Yohann Robert[4], Xavier Laval[2], Tanguy Laurencin[1], Philippe Chaffanjon[2,4], Barbara Fayard[5], and Elodie Boller[6]

[1]Univ. Grenoble Alpes, CNRS, Grenoble INP, 3SR, Grenoble, F-38000, France
[2]Univ. Grenoble Alpes, CNRS, Grenoble INP, GIPSA-lab, Grenoble, F-38000, France
[3]Univ. Grenoble Alpes, CHU Grenoble Alpes, CNRS, Grenoble INP, IAB, Grenoble, F-38000, France
[4]Univ. Grenoble Alpes, CHU Grenoble Alpes, LADAF, Grenoble, F-38000, France
[5]Novitom, Grenoble, F-38000, France
[6]ID19 beamline, ESRF - European Synchrotron Radiation Facility, CS40220, Grenoble 38043, France

SUPPLEMENTARY INFORMATION

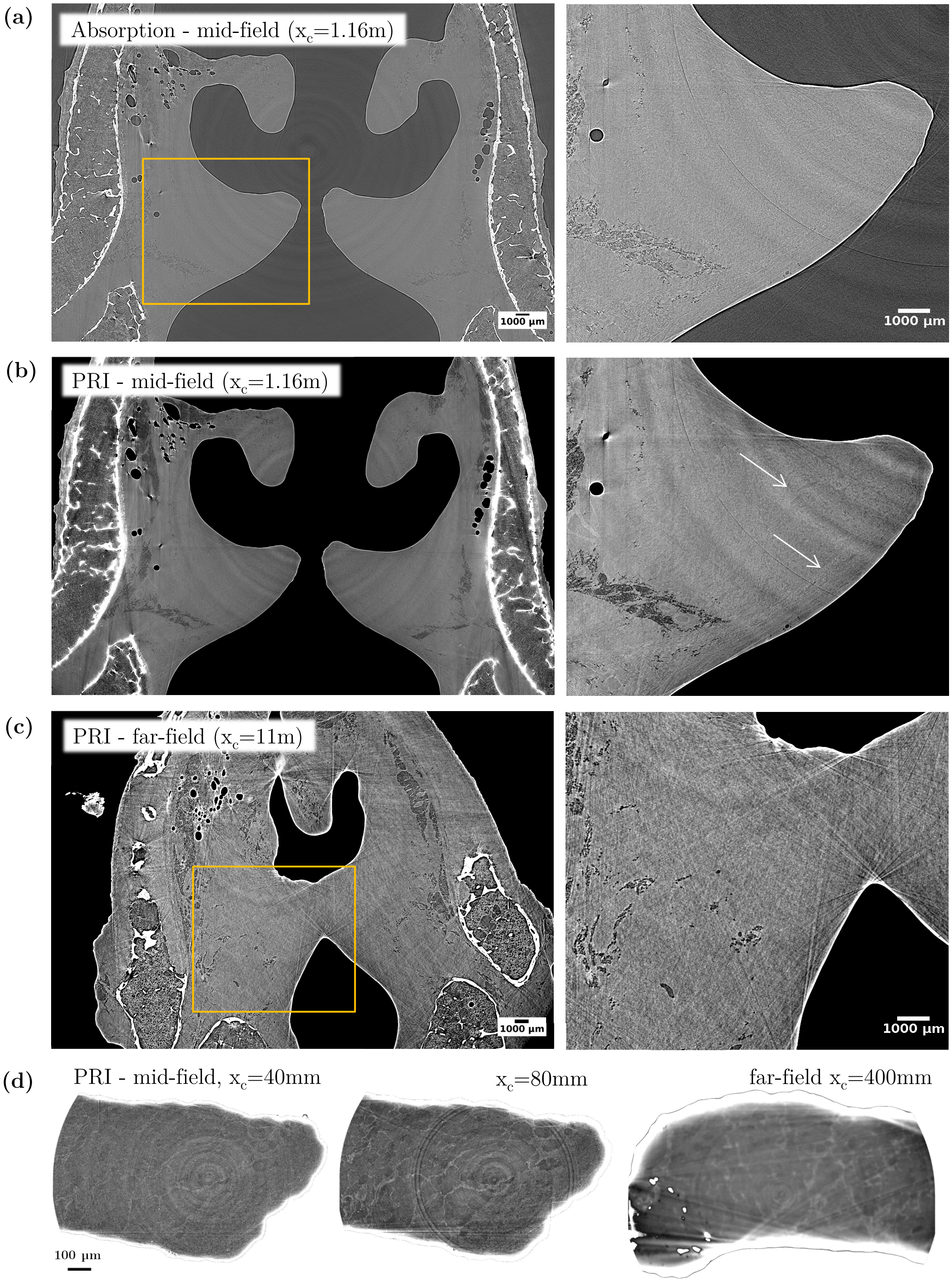

Figure S1. Influence of various optical settings on synchrotron X-ray microtomographic images of the database, reconstructed in coronal plane. Experiments on larynges (a), (b) L_8 and (c) L_9; on a vocal-fold sample (d) L_{10}-S_1.

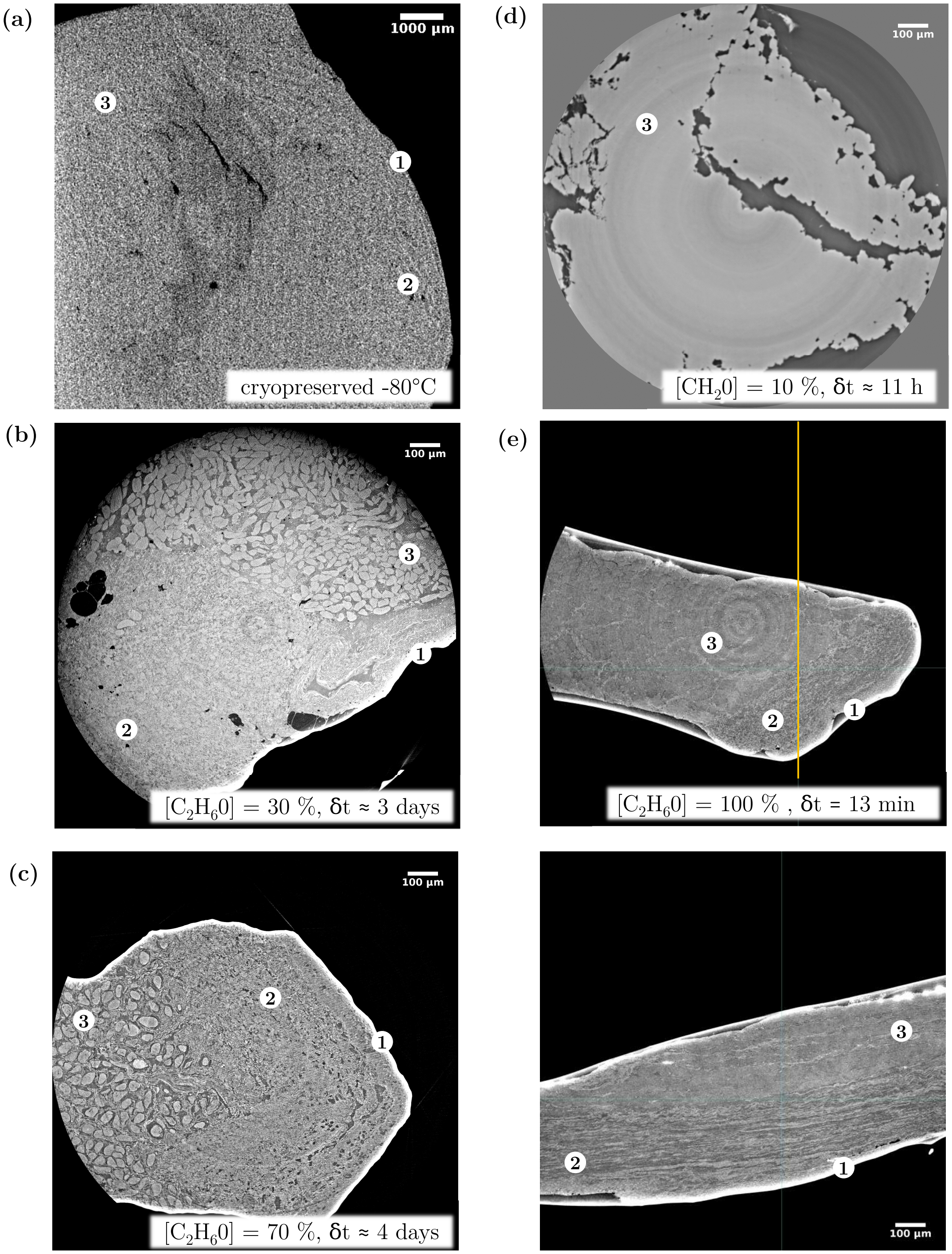

Figure S2. Influence of various conditions of tissue conservation and contrast agents on mid-field X-ray microtomographic images of the database. Coronal views of vocal-fold samples (a) L_2-S_1, (b) L_6-S_1, (c) L_{10}-S_3, (d) L_7-S_1. Coronal and perpendicular view (along yellow line) of the vocal-fold sample (e) L_{10}-S_4. ① EP, ② LP, ③ M.

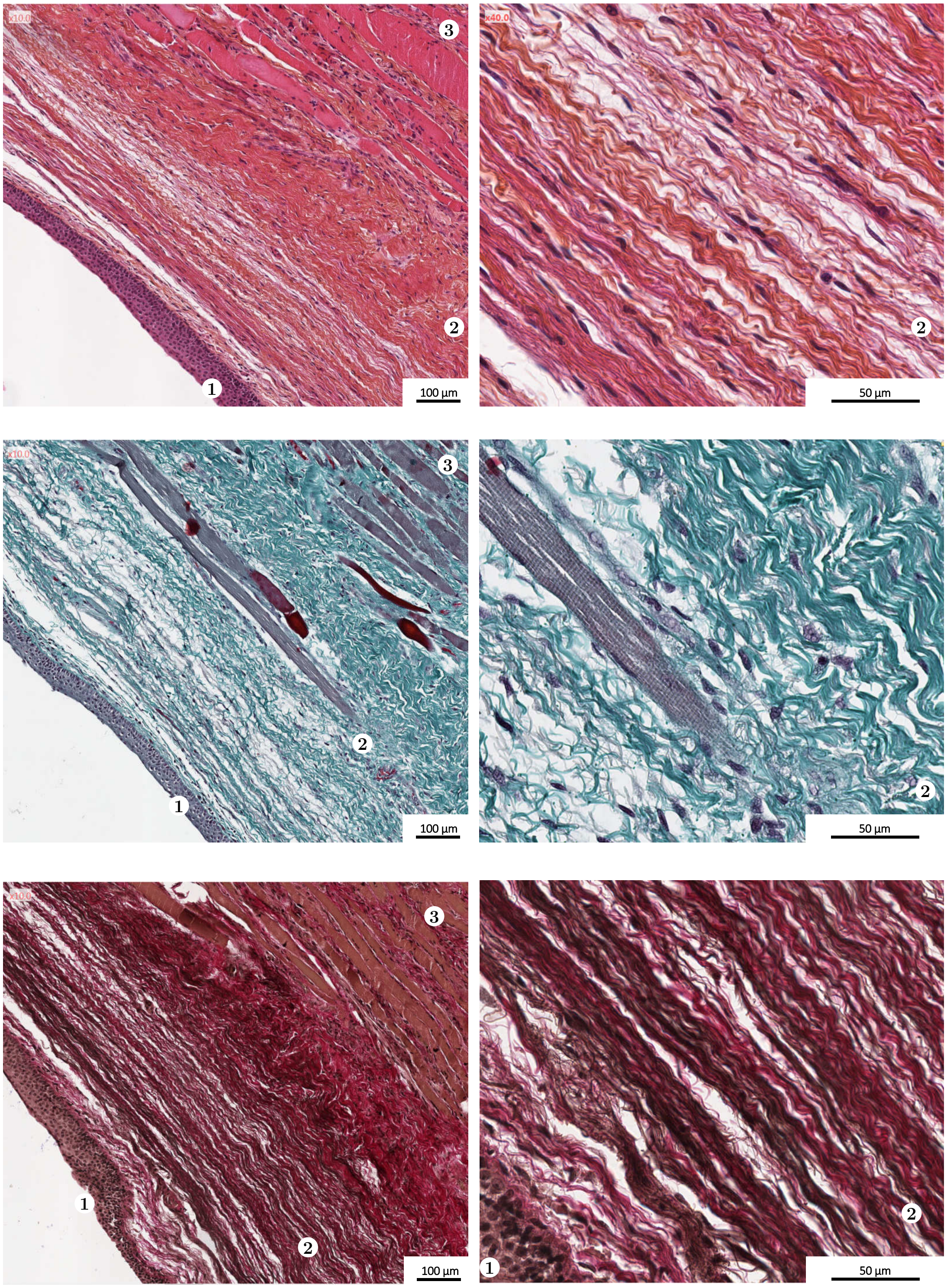

Figure S3. Histological photomicrographs of a 2D transversal view of L_{10}-S_5. *(top)* HES stain: collagen fibers (yellow-orange); cytoplasms, striated muscular and elastin fibers (pink); *nuclei* (blue-purple). *(middle)* Masson Trichrome stain: collagen fibers (green); cytoplasms and striated muscular (pink); elastin fibers (gray); *nuclei* (blue-purple). *(bottom)* Elastin stain: elastin fibers (black); ① EP, ② LP, ③ M.

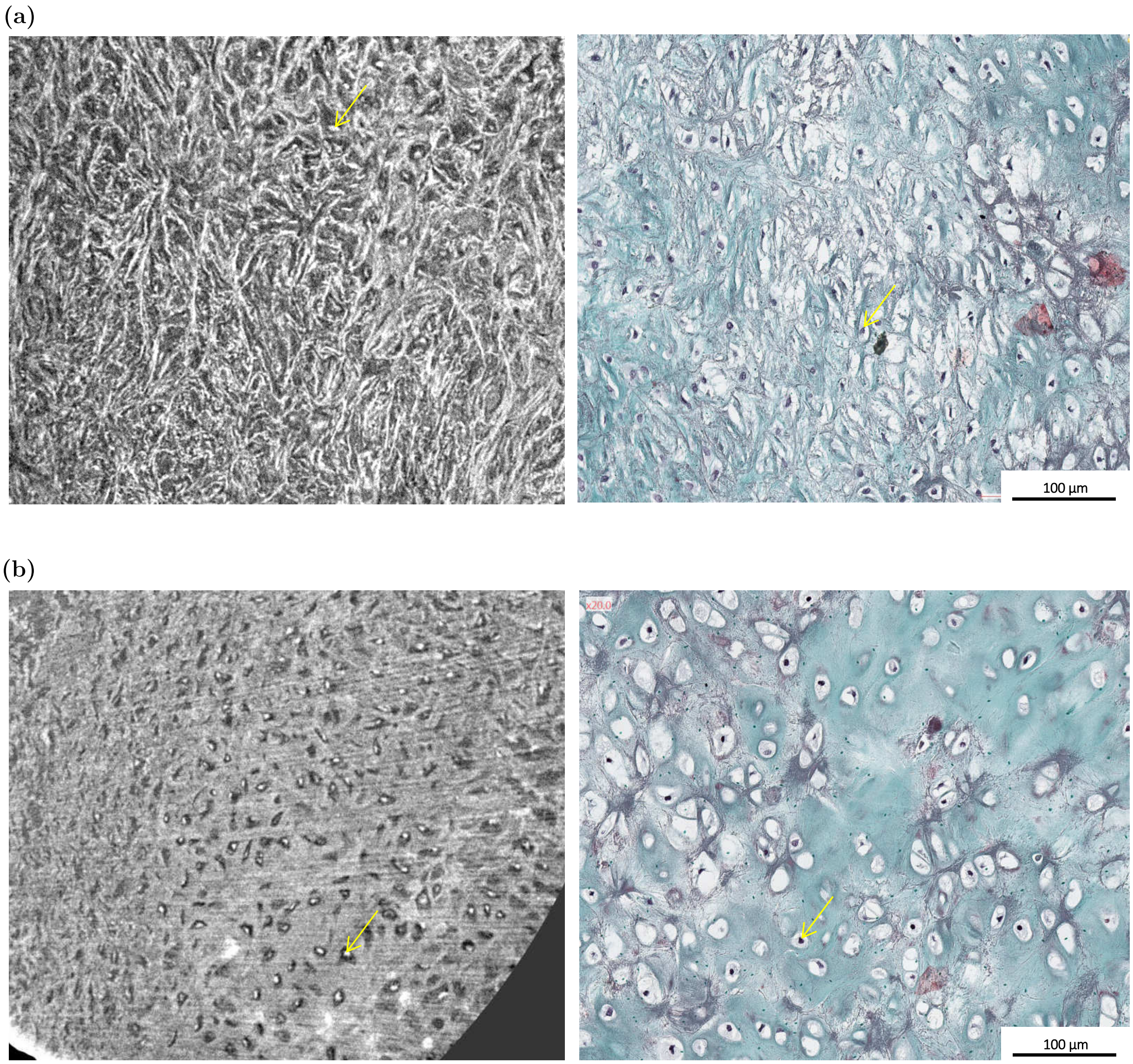

Figure S4. Comparison between (a) elastic arytenoid cartilage and (b) hyaline arytenoid cartilage acquired by two imaging modes: *(left)* 2D coronal views of L_{10}-S_3 ($[C_2H_60]$ = 70 %), reconstructed from synchrotron high-resolution X-ray microtomographic images (PRI mode); *(right)* 2D histological photomicrographs of L_{10}-S_5. Arrows: chondrocyte (*nucleus*) in *lacuna*.

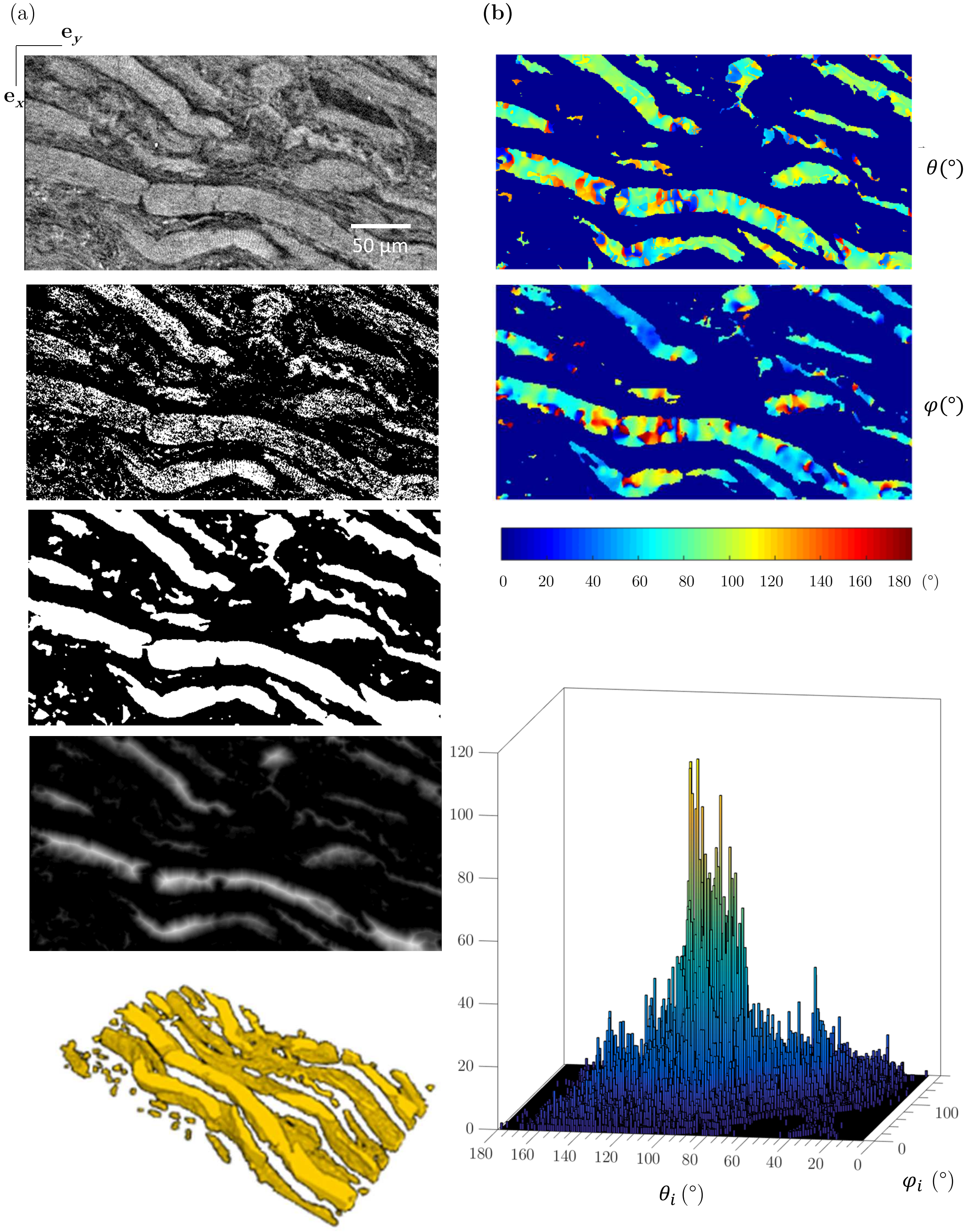

Figure S5. Typical quantitative processing of *vocalis* images : (a) Pre-processing operations applied to the images before running 3D orientation algorithms. (b) 3D angular maps and distribution obtained after 3D orientation analyses processed within a whole volume, and displayed for a single slice.

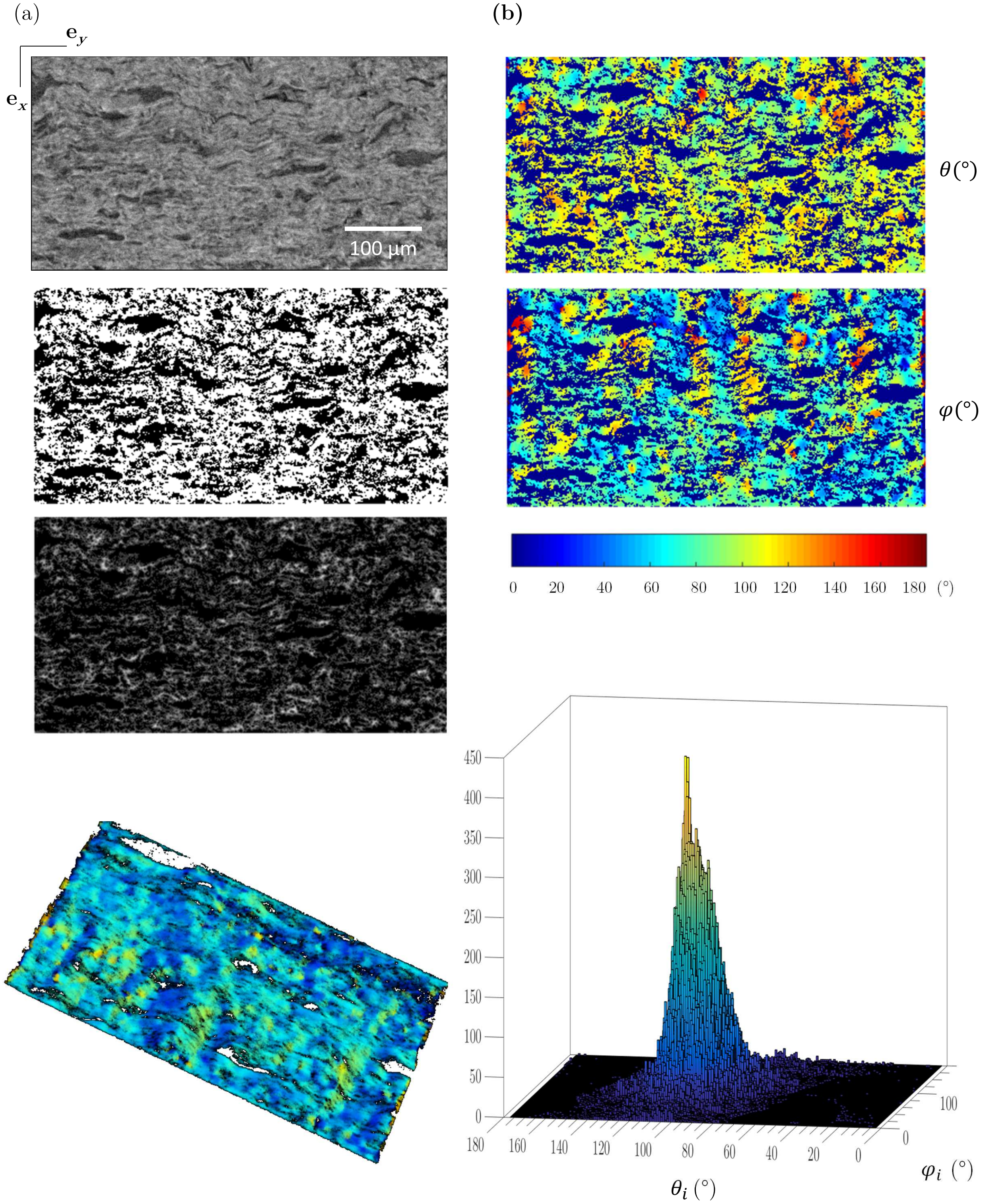

Figure S6. Typical quantitative processing of *lamina propria* images : (a) Pre-processing operations applied to the images before running 3D orientation algorithms. (b) 3D angular maps and distribution obtained after 3D orientation analyses processed within a whole volume, and displayed for a single slice.

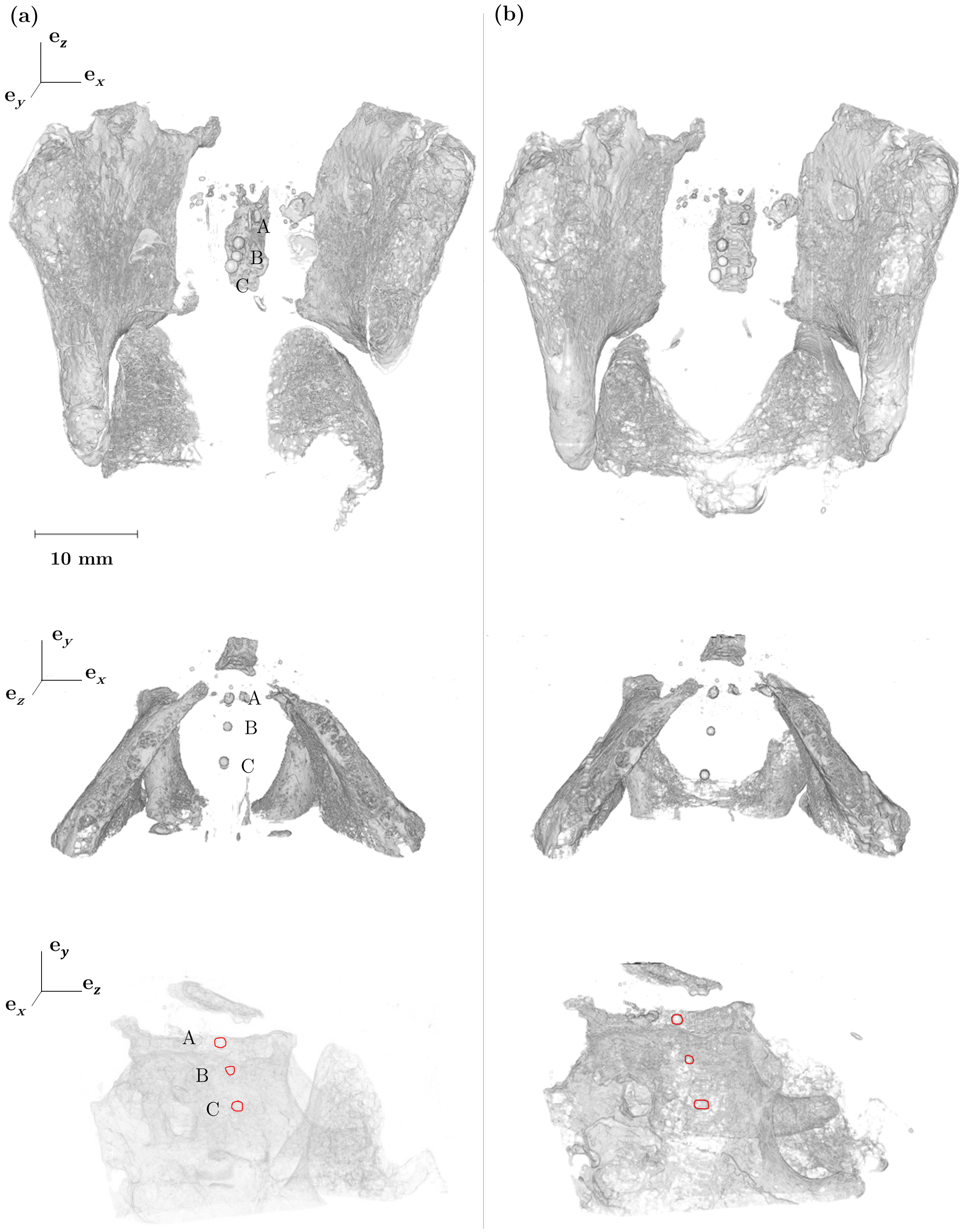

Figure S7. 3D reconstructions of larynx L_6 (a) at rest and (b) at the maximal macroscopic stretch of the vocal folds achieved by cricothyroid approximation.

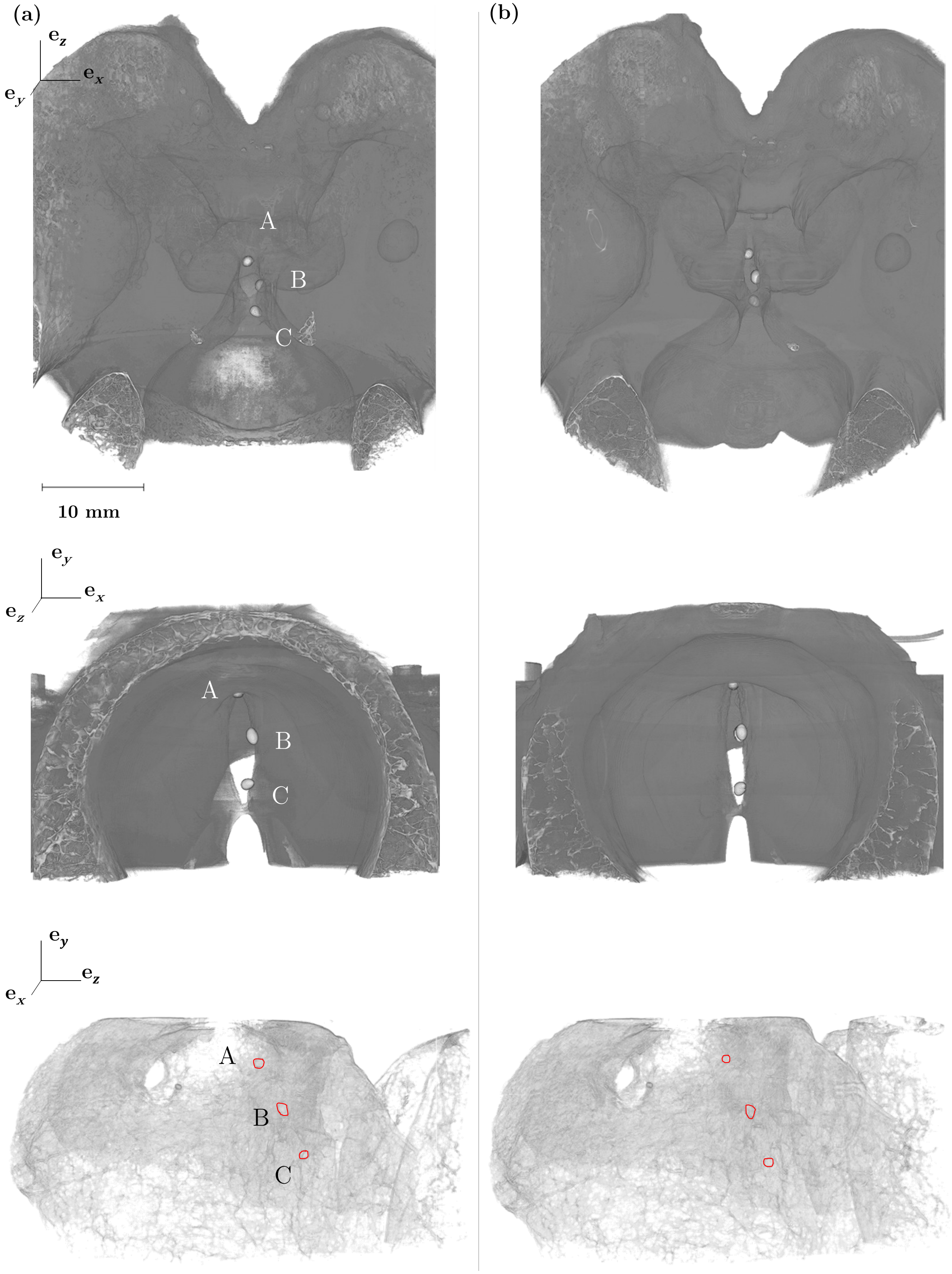

Figure S8. 3D reconstructions of larynx L_8 (a) at rest and (b) at the maximal macroscopic stretch of the vocal folds achieved by cricothyroid approximation.
